# Supplementary material for: Metabolomic changes associated with frontotemporal lobar degeneration syndromes
Source: J Neurol. 2020 Apr 10;267(8):2228–38. doi: 10.1007/s00415-020-09824-1 (PMC7359154; doi:10.1007/s00415-020-09824-1)
Supplement: Supplementary file 1 — Supplementary file1 (PDF 840 kb) [file 415_2020_9824_MOESM1_ESM.pdf]

Biochemical	Subpathway	Superpathway	Human Metabolome Database ID	Fold change (FTLD vs Control)	FDR corrected p value
Alanine	Alanine And Aspartate Metabolism	Amino Acid	HMDB00161	1.06	0.4175
Asparagine	Alanine And Aspartate Metabolism	Amino Acid	HMDB00168	0.95	0.3124
Aspartate	Alanine And Aspartate Metabolism	Amino Acid	HMDB00191	1.36	0.0301
N-Acetylalanine	Alanine And Aspartate Metabolism	Amino Acid	HMDB00766	1.09	0.2334
N-Acetylaspartate (NAA)	Alanine And Aspartate Metabolism	Amino Acid	HMDB00812	0.97	0.8031
N-Carbamoylalanine	Alanine And Aspartate Metabolism	Amino Acid		1.19	0.5733
N-Methylalanine	Alanine And Aspartate Metabolism	Amino Acid	HMDB01906	1.48	0.4175
Creatine	Creatine Metabolism	Amino Acid	HMDB00064	0.96	0.8218
Creatinine	Creatine Metabolism	Amino Acid	HMDB00562	1.06	0.3832
Guanidinoacetate	Creatine Metabolism	Amino Acid	HMDB00128	0.72	0.0000
4-Hydroxyglutamate	Glutamate Metabolism	Amino Acid	HMDB01344	1.65	0.4639
Beta-Citrylglutamate	Glutamate Metabolism	Amino Acid		1.54	0.0001
Carboxyethyl-Gaba	Glutamate Metabolism	Amino Acid	HMDB02201	0.75	0.0981
Glutamate	Glutamate Metabolism	Amino Acid	HMDB00148	1.18	0.3939
Glutamine	Glutamate Metabolism	Amino Acid	HMDB00641	1.01	0.8696
N-Acetylglutamate	Glutamate Metabolism	Amino Acid	HMDB01138	1.03	0.8547
N-Acetylglutamine	Glutamate Metabolism	Amino Acid	HMDB06029	1.13	0.5802
Pyroglutamine*	Glutamate Metabolism	Amino Acid		1.54	0.0489
S-1-Pyrroline-5-Carboxylate	Glutamate Metabolism	Amino Acid	HMDB01301	1.61	0.0021
2-Aminobutyrate	Glutathione Metabolism	Amino Acid	HMDB00650	0.81	0.0029
2-Hydroxybutyrate/2-Hydroxyisobutyrate	Glutathione Metabolism	Amino Acid		0.89	0.4175
5-Oxoproline	Glutathione Metabolism	Amino Acid	HMDB00267	0.99	0.9268
Cys-Gly, Oxidized	Glutathione Metabolism	Amino Acid		0.82	0.4175
Cysteine-Glutathione Disulfide	Glutathione Metabolism	Amino Acid	HMDB00656	1.21	0.3885
Cysteinylglycine	Glutathione Metabolism	Amino Acid	HMDB00078	0.90	0.6966
2-Methylserine	Glycine, Serine And Threonine Metabolism	Amino Acid		0.51	0.0000
Betaine	Glycine, Serine And Threonine Metabolism	Amino Acid	HMDB00043	0.94	0.4686

Dimethylglycine	Glycine, Serine And Threonine Metabolism	Amino Acid	HMDB00092	1.05	0.6877
Glycine	Glycine, Serine And Threonine Metabolism	Amino Acid	HMDB00123	1.06	0.5066
N-Acetylglycine	Glycine, Serine And Threonine Metabolism	Amino Acid	HMDB00532	0.78	0.2337
N-Acetylserine	Glycine, Serine And Threonine Metabolism	Amino Acid	HMDB02931	1.12	0.2722
N-Acetylthreonine	Glycine, Serine And Threonine Metabolism	Amino Acid		1.19	0.1988
Sarcosine	Glycine, Serine And Threonine Metabolism	Amino Acid	HMDB00271	0.77	0.0000
Serine	Glycine, Serine And Threonine Metabolism	Amino Acid	HMDB00187	0.94	0.2784
Threonine	Glycine, Serine And Threonine Metabolism	Amino Acid	HMDB00167	0.98	0.8349
4-Guanidinobutanoate	Guanidino And Acetamido Metabolism	Amino Acid	HMDB03464	1.38	0.3334
1-Methylhistidine	Histidine Metabolism	Amino Acid	HMDB00001	1.16	0.4858
1-Methylimidazoleacetate	Histidine Metabolism	Amino Acid	HMDB02820	1.32	0.2294
3-Methylhistidine	Histidine Metabolism	Amino Acid	HMDB00479	0.91	0.8417
Formiminoglutamate	Histidine Metabolism	Amino Acid	HMDB00854	1.10	0.6435
Histidine	Histidine Metabolism	Amino Acid	HMDB00177	0.98	0.7104
Hydantoin-5-Propionic Acid	Histidine Metabolism	Amino Acid	HMDB01212	1.36	0.3002
Imidazole Lactate	Histidine Metabolism	Amino Acid	HMDB02320	0.94	0.5629
Imidazole Propionate	Histidine Metabolism	Amino Acid	HMDB02271	2.41	0.1909
N-Acetyl-1-Methylhistidine*	Histidine Metabolism	Amino Acid		1.25	0.8090
N-Acetyl-3-Methylhistidine*	Histidine Metabolism	Amino Acid		0.78	0.5468
N-Acetylcarnosine	Histidine Metabolism	Amino Acid	HMDB12881	0.86	0.3832
N-Acetylhistidine	Histidine Metabolism	Amino Acid	HMDB32055	1.16	0.4377
2,3-Dihydroxy-2-Methylbutyrate	Leucine, Isoleucine And Valine Metabolism	Amino Acid	HMDB29576	1.08	0.6905
2-Hydroxy-3-Methylvalerate	Leucine, Isoleucine And Valine Metabolism	Amino Acid	HMDB00317	1.02	0.9178
2-Methylbutyrylcarnitine (C5)	Leucine, Isoleucine And Valine Metabolism	Amino Acid	HMDB00378	0.99	0.9390

3-Hydroxy-2-Ethylpropionate	Leucine, Isoleucine And Valine Metabolism	Amino Acid	HMDB00396	0.99	0.8961
3-Hydroxyisobutyrate	Leucine, Isoleucine And Valine Metabolism	Amino Acid	HMDB00336	0.88	0.4686
3-Methyl-2-Oxobutyrate	Leucine, Isoleucine And Valine Metabolism	Amino Acid	HMDB00019	0.90	0.1584
3-Methyl-2-Oxovalerate	Leucine, Isoleucine And Valine Metabolism	Amino Acid	HMDB03736	0.94	0.4817
3-Methylglutaconate	Leucine, Isoleucine And Valine Metabolism	Amino Acid	HMDB00522	1.14	0.6562
3-Methylglutarylcarnitine (2)	Leucine, Isoleucine And Valine Metabolism	Amino Acid	HMDB00552	1.43	0.2743
4-Methyl-2-Oxopentanoate	Leucine, Isoleucine And Valine Metabolism	Amino Acid	HMDB00695	0.89	0.1886
Alpha-Hydroxyisocaproate	Leucine, Isoleucine And Valine Metabolism	Amino Acid	HMDB00746	0.94	0.6074
Alpha-Hydroxyisovalerate	Leucine, Isoleucine And Valine Metabolism	Amino Acid	HMDB00407	0.96	0.8128
Beta-Hydroxyisovalerate	Leucine, Isoleucine And Valine Metabolism	Amino Acid	HMDB00754	1.05	0.7330
Ethylmalonate	Leucine, Isoleucine And Valine Metabolism	Amino Acid	HMDB00622	0.76	0.3929
Isobutyrylcarnitine (C4)	Leucine, Isoleucine And Valine Metabolism	Amino Acid	HMDB00736	1.06	0.7686
Isobutyrylglycine	Leucine, Isoleucine And Valine Metabolism	Amino Acid	HMDB00730	1.06	0.8417
Isoleucine	Leucine, Isoleucine And Valine Metabolism	Amino Acid	HMDB00172	1.05	0.5026
Isovalerate (I5:0)	Leucine, Isoleucine And Valine Metabolism	Amino Acid	HMDB00718	1.04	0.8090
Isovalerylcarnitine (C5)	Leucine, Isoleucine And Valine Metabolism	Amino Acid	HMDB00688	0.88	0.4686
Isovalerylglycine	Leucine, Isoleucine And Valine Metabolism	Amino Acid	HMDB00678	0.97	0.8854
Leucine	Leucine, Isoleucine And Valine Metabolism	Amino Acid	HMDB00687	1.03	0.6444
N-Acetylisoleucine	Leucine, Isoleucine And Valine Metabolism	Amino Acid	HMDB61684	1.26	0.4817

N-Acetylleucine	Leucine, Isoleucine And Valine Metabolism	Amino Acid	HMDB11756	1.03	0.8696
N-Acetylvaline	Leucine, Isoleucine And Valine Metabolism	Amino Acid	HMDB11757	1.08	0.3832
Tiglylcarnitine (C5:1-Dc)	Leucine, Isoleucine And Valine Metabolism	Amino Acid	HMDB02366	0.82	0.0595
Valine	Leucine, Isoleucine And Valine Metabolism	Amino Acid	HMDB00883	1.01	0.9490
2-Aminoadipate	Lysine Metabolism	Amino Acid	HMDB00510	0.80	0.2334
5-(Galactosylhydroxy)-L-Lysine	Lysine Metabolism	Amino Acid		1.34	0.0362
5-Hydroxylysine	Lysine Metabolism	Amino Acid	HMDB00450	1.33	0.0886
6-Oxopiperidine-2-Carboxylate	Lysine Metabolism	Amino Acid	HMDB61705	1.07	0.6282
Glutarylcarnitine (C5-Dc)	Lysine Metabolism	Amino Acid	HMDB13130	1.21	0.2077
Lysine	Lysine Metabolism	Amino Acid	HMDB00182	0.98	0.6346
N-Trimethyl 5-Aminovalerate	Lysine Metabolism	Amino Acid		1.07	0.6595
N6,N6,N6-Trimethyllysine	Lysine Metabolism	Amino Acid	HMDB01325	1.07	0.7133
N6-Acetyllysine	Lysine Metabolism	Amino Acid	HMDB00206	1.06	0.3972
Pipecolate	Lysine Metabolism	Amino Acid	HMDB00070	0.92	0.6904
Alpha-Ketobutyrate	Methionine, Cysteine, Sam And Taurine Metabolism	Amino Acid	HMDB00005	0.41	0.0000
Cystathionine	Methionine, Cysteine, Sam And Taurine Metabolism	Amino Acid	HMDB00099	0.87	0.6565
Cysteine	Methionine, Cysteine, Sam And Taurine Metabolism	Amino Acid	HMDB00574	1.11	0.2471
Cysteine S-Sulfate	Methionine, Cysteine, Sam And Taurine Metabolism	Amino Acid	HMDB00731	0.86	0.3134
Cysteine Sulfinic Acid	Methionine, Cysteine, Sam And Taurine Metabolism	Amino Acid	HMDB00996	1.00	0.9921
Cystine	Methionine, Cysteine, Sam And Taurine Metabolism	Amino Acid	HMDB00192	1.14	0.1842
Hypotaurine	Methionine, Cysteine, Sam And Taurine Metabolism	Amino Acid	HMDB00965	2.26	0.0000
Methionine	Methionine, Cysteine, Sam And Taurine Metabolism	Amino Acid	HMDB00696	1.01	0.9398
Methionine Sulfone	Methionine, Cysteine, Sam And Taurine Metabolism	Amino Acid		1.28	0.0403

Methionine Sulfoxide	Methionine, Cysteine, Sam And Taurine Metabolism	Amino Acid	HMDB02005	1.20	0.2019
N-Acetylmethionine	Methionine, Cysteine, Sam And Taurine Metabolism	Amino Acid	HMDB11745	1.45	0.0053
N-Acetyltaurine	Methionine, Cysteine, Sam And Taurine Metabolism	Amino Acid		1.22	0.1654
N-Formylmethionine	Methionine, Cysteine, Sam And Taurine Metabolism	Amino Acid	HMDB01015	1.14	0.1909
N-Methyltaurine	Methionine, Cysteine, Sam And Taurine Metabolism	Amino Acid		1.40	0.5379
S-Adenosylhomocysteine (Sah)	Methionine, Cysteine, Sam And Taurine Metabolism	Amino Acid	HMDB00939	1.24	0.1308
S-Methylcysteine	Methionine, Cysteine, Sam And Taurine Metabolism	Amino Acid	HMDB02108	0.97	0.8854
S-Methylcysteine Sulfoxide	Methionine, Cysteine, Sam And Taurine Metabolism	Amino Acid	HMDB29432	1.00	0.9921
S-Methylmethionine	Methionine, Cysteine, Sam And Taurine Metabolism	Amino Acid	HMDB38670	0.82	0.7205
Taurine	Methionine, Cysteine, Sam And Taurine Metabolism	Amino Acid	HMDB00251	1.72	0.0000
4-Hydroxyphenylacetate	Phenylalanine Metabolism	Amino Acid	HMDB00020	1.27	0.3387
N-Acetylphenylalanine	Phenylalanine Metabolism	Amino Acid	HMDB00512	1.11	0.6435
Phenylacetate	Phenylalanine Metabolism	Amino Acid	HMDB00209	1.36	0.1545
Phenylalanine	Phenylalanine Metabolism	Amino Acid	HMDB00159	1.06	0.2256
Phenyllactate (Pla)	Phenylalanine Metabolism	Amino Acid	HMDB00779	1.11	0.4955
Phenylpyruvate	Phenylalanine Metabolism	Amino Acid	HMDB00205	1.06	0.6565
(N(1) + N(8))-Acetylspermidine	Polyamine Metabolism	Amino Acid		1.31	0.0303
4-Acetamidobutanoate	Polyamine Metabolism	Amino Acid	HMDB03681	1.27	0.1273
5-Methylthioadenosine (Mta)	Polyamine Metabolism	Amino Acid	HMDB01173	1.30	0.0031
Acisoga	Polyamine Metabolism	Amino Acid		1.19	0.4175
N-Acetylputrescine	Polyamine Metabolism	Amino Acid	HMDB02064	1.21	0.0168
Spermidine	Polyamine Metabolism	Amino Acid	HMDB01257	4.38	0.0001
3-Indoxyl Sulfate	Tryptophan Metabolism	Amino Acid	HMDB00682	1.21	0.2779
5-Bromotryptophan	Tryptophan Metabolism	Amino Acid		1.02	0.8349
5-Hydroxyindoleacetate	Tryptophan Metabolism	Amino Acid	HMDB00763	1.43	0.3157
Anthranilate	Tryptophan Metabolism	Amino Acid	HMDB01123	1.19	0.2264

C-Glycosyltryptophan	Tryptophan Metabolism	Amino Acid		1.26	0.0959
Indole-3-Carboxylic Acid	Tryptophan Metabolism	Amino Acid	HMDB03320	1.38	0.1911
Indoleacetate	Tryptophan Metabolism	Amino Acid	HMDB00197	1.07	0.7497
Indoleacetylglutamine	Tryptophan Metabolism	Amino Acid	HMDB13240	1.30	0.6031
Indolelactate	Tryptophan Metabolism	Amino Acid	HMDB00671	0.95	0.6586
Indolepropionate	Tryptophan Metabolism	Amino Acid	HMDB02302	0.89	0.5779
Kynurenate	Tryptophan Metabolism	Amino Acid	HMDB00715	0.92	0.4727
Kynurenine	Tryptophan Metabolism	Amino Acid	HMDB00684	1.13	0.1613
N-Acetylkynurenine (2)	Tryptophan Metabolism	Amino Acid		1.03	0.9451
N-Acetyltryptophan	Tryptophan Metabolism	Amino Acid	HMDB13713	0.97	0.8368
N-Formylanthranilic Acid	Tryptophan Metabolism	Amino Acid	HMDB04089	1.23	0.4686
Picolinate	Tryptophan Metabolism	Amino Acid	HMDB02243	0.92	0.6586
Serotonin	Tryptophan Metabolism	Amino Acid	HMDB00259	91.89	0.0000
Tryptophan	Tryptophan Metabolism	Amino Acid	HMDB00929	0.95	0.4161
Tryptophan Betaine	Tryptophan Metabolism	Amino Acid	HMDB61115	0.43	0.0033
Xanthurenate	Tryptophan Metabolism	Amino Acid	HMDB00881	0.78	0.1637
2-Hydroxyphenylacetate	Tyrosine Metabolism	Amino Acid	HMDB00669	1.35	0.1373
3-(4-Hydroxyphenyl)Lactate	Tyrosine Metabolism	Amino Acid	HMDB00755	0.91	0.4348
3-Methoxytyramine Sulfate	Tyrosine Metabolism	Amino Acid		9.41	0.3134
3-Methoxytyrosine	Tyrosine Metabolism	Amino Acid	HMDB01434	28.15	0.2824
4-Hydroxyphenylpyruvate	Tyrosine Metabolism	Amino Acid	HMDB00707	1.14	0.2775
Catechol Glucuronide	Tyrosine Metabolism	Amino Acid		Excluded	Excluded
Dopamine 3-O-Sulfate	Tyrosine Metabolism	Amino Acid	HMDB06275	2.92	0.1842
Dopamine 4-Sulfate	Tyrosine Metabolism	Amino Acid	HMDB04148	3.72	0.1969
Gentisate	Tyrosine Metabolism	Amino Acid	HMDB00152	1.06	0.9173
Homovanillate (Hva)	Tyrosine Metabolism	Amino Acid	HMDB00118	2.33	0.3144
N-Acetyltyrosine	Tyrosine Metabolism	Amino Acid	HMDB00866	0.89	0.4377
N-Formylphenylalanine	Tyrosine Metabolism	Amino Acid		0.96	0.8738
P-Cresol-Glucuronide*	Tyrosine Metabolism	Amino Acid	HMDB11686	1.84	0.0937
Phenol Glucuronide	Tyrosine Metabolism	Amino Acid	HMDB60014	Excluded	Excluded
Phenol Sulfate	Tyrosine Metabolism	Amino Acid	HMDB60015	1.37	0.3796
Thyroxine	Tyrosine Metabolism	Amino Acid	HMDB01918	1.04	0.6448
Tyramine O-Sulfate	Tyrosine Metabolism	Amino Acid	HMDB06409	1.74	0.5236

Tyrosine	Tyrosine Metabolism	Amino Acid	HMDB00158	0.99	0.9286
Vanillactate	Tyrosine Metabolism	Amino Acid	HMDB00913	20.64	0.3367
Vanillic Alcohol Sulfate	Tyrosine Metabolism	Amino Acid		Excluded	Excluded
Vanillylmandelate (Vma)	Tyrosine Metabolism	Amino Acid	HMDB00291	1.19	0.1496
2-Oxoarginine*	Urea Cycle; Arginine And Proline Metabolism	Amino Acid	HMDB04225	0.96	0.8449
Argininate*	Urea Cycle; Arginine And Proline Metabolism	Amino Acid	HMDB03148	0.87	0.4377
Arginine	Urea Cycle; Arginine And Proline Metabolism	Amino Acid	HMDB00517	1.04	0.6915
Citrulline	Urea Cycle; Arginine And Proline Metabolism	Amino Acid	HMDB00904	0.92	0.3193
Dimethylarginine (Sdma + Adma)	Urea Cycle; Arginine And Proline Metabolism	Amino Acid	HMDB01539	1.09	0.1081
Homoarginine	Urea Cycle; Arginine And Proline Metabolism	Amino Acid	HMDB00670	0.83	0.0192
Homocitrulline	Urea Cycle; Arginine And Proline Metabolism	Amino Acid	HMDB00679	1.35	0.3308
N-Acetylarginine	Urea Cycle; Arginine And Proline Metabolism	Amino Acid	HMDB04620	0.94	0.7463
N-Acetylcitrulline	Urea Cycle; Arginine And Proline Metabolism	Amino Acid	HMDB00856	1.09	0.8260
N-Acetylproline	Urea Cycle; Arginine And Proline Metabolism	Amino Acid		1.16	0.6267
N-Delta-Acetylornithine	Urea Cycle; Arginine And Proline Metabolism	Amino Acid		1.00	0.9887
N-Methylproline	Urea Cycle; Arginine And Proline Metabolism	Amino Acid		0.87	0.8334
N2,N5-Diacetylornithine	Urea Cycle; Arginine And Proline Metabolism	Amino Acid		0.91	0.8090
Ornithine	Urea Cycle; Arginine And Proline Metabolism	Amino Acid	HMDB03374	0.95	0.5938
Pro-Hydroxy-Pro	Urea Cycle; Arginine And Proline Metabolism	Amino Acid	HMDB06695	1.55	0.0032
Proline	Urea Cycle; Arginine And Proline Metabolism	Amino Acid	HMDB00162	1.14	0.0910

Trans-4-Hydroxyproline	Urea Cycle; Arginine And Proline Metabolism	Amino Acid	HMDB00725	1.20	0.1830
Urea	Urea Cycle; Arginine And Proline Metabolism	Amino Acid	HMDB00294	1.15	0.3106
N6-Carboxymethyllysine	Advanced Glycation End-Product	Carbohydrate		1.26	0.4534
Erythronate*	Aminosugar Metabolism	Carbohydrate	HMDB00613	1.16	0.1654
Glucuronate	Aminosugar Metabolism	Carbohydrate	HMDB00127	1.16	0.4377
N-Acetylglucosamine/N-Acetylgalactosamine	Aminosugar Metabolism	Carbohydrate	HMDB00215	1.12	0.2334
N-Acetylglucosaminylasparagine	Aminosugar Metabolism	Carbohydrate	HMDB00489	1.85	0.0009
N-Acetylneuraminate	Aminosugar Metabolism	Carbohydrate	HMDB00230	1.57	0.0000
Sucrose	Disaccharides And Oligosaccharides	Carbohydrate	HMDB00258	3.24	0.0403
Fructose	Fructose, Mannose And Galactose Metabolism	Carbohydrate	HMDB00660	1.10	0.6465
Mannitol/Sorbitol	Fructose, Mannose And Galactose Metabolism	Carbohydrate	HMDB00247	0.98	0.9792
Mannose	Fructose, Mannose And Galactose Metabolism	Carbohydrate	HMDB00169	1.03	0.8589
Maltose	Glycogen Metabolism	Carbohydrate	HMDB00163	3.65	0.0000
Maltotetraose	Glycogen Metabolism	Carbohydrate	HMDB01296	29.70	0.0000
Maltotriose	Glycogen Metabolism	Carbohydrate	HMDB01262	31.14	0.0000
1,5-Anhydroglucitol (1,5-Ag)	Glycolysis, Gluconeogenesis, And Pyruvate Metabolism	Carbohydrate	HMDB02712	0.99	0.9286
3-Phosphoglycerate	Glycolysis, Gluconeogenesis, And Pyruvate Metabolism	Carbohydrate	HMDB00807	1.81	0.5066
Glucose	Glycolysis, Gluconeogenesis, And Pyruvate Metabolism	Carbohydrate	HMDB00122	1.07	0.4175
Glycerate	Glycolysis, Gluconeogenesis, And Pyruvate Metabolism	Carbohydrate	HMDB00139	0.95	0.5624
Lactate	Glycolysis, Gluconeogenesis, And Pyruvate Metabolism	Carbohydrate	HMDB00190	1.26	0.1545
Pyruvate	Glycolysis, Gluconeogenesis, And Pyruvate Metabolism	Carbohydrate	HMDB00243	0.66	0.0033
Arabinose	Pentose Metabolism	Carbohydrate	HMDB00646	1.13	0.5343
Arabitol/Xylitol	Pentose Metabolism	Carbohydrate		0.99	0.9592
Arabonate/Xylonate	Pentose Metabolism	Carbohydrate		1.04	0.8761

Ribitol	Pentose Metabolism	Carbohydrate	HMDB00508	1.06	0.6001
Ribonate	Pentose Metabolism	Carbohydrate	HMDB00867	1.09	0.4727
Xylose	Pentose Metabolism	Carbohydrate	HMDB00098	1.07	0.8353
Gulonate*	Ascorbate And Aldarate Metabolism	Cofactors and Vitamins	HMDB03290	1.34	0.2277
Oxalate (Ethanedioate)	Ascorbate And Aldarate Metabolism	Cofactors and Vitamins	HMDB02329	0.83	0.1988
Threonate	Ascorbate And Aldarate Metabolism	Cofactors and Vitamins	HMDB00943	0.74	0.0192
Folate	Folate Metabolism	Cofactors and Vitamins	HMDB00121	Excluded	Excluded
Bilirubin (E,E)*	Hemoglobin And Porphyrin Metabolism	Cofactors and Vitamins		0.98	0.8996
Bilirubin (E,Z Or Z,E)*	Hemoglobin And Porphyrin Metabolism	Cofactors and Vitamins	HMDB00488	1.00	0.9833
Bilirubin (Z,Z)	Hemoglobin And Porphyrin Metabolism	Cofactors and Vitamins	HMDB00054	0.94	0.6579
Biliverdin	Hemoglobin And Porphyrin Metabolism	Cofactors and Vitamins	HMDB01008	1.02	0.9445
I-Urobilinogen	Hemoglobin And Porphyrin Metabolism	Cofactors and Vitamins	HMDB04157	Excluded	Excluded
1-Methylnicotinamide	Nicotinate And Nicotinamide Metabolism	Cofactors and Vitamins	HMDB00699	0.93	0.6465
Adenosine 5'-Diphosphoribose (Adp-Ribose)	Nicotinate And Nicotinamide Metabolism	Cofactors and Vitamins	HMDB01178	6.14	0.0001
N1-Methyl-2-Pyridone-5-Carboxamide	Nicotinate And Nicotinamide Metabolism	Cofactors and Vitamins	HMDB04193	1.14	0.6595
N1-Methyl-4-Pyridone-3-Carboxamide	Nicotinate And Nicotinamide Metabolism	Cofactors and Vitamins	HMDB04194	1.12	0.7497
Nicotinamide	Nicotinate And Nicotinamide Metabolism	Cofactors and Vitamins	HMDB01406	2.26	0.0000
Quinolinate	Nicotinate And Nicotinamide Metabolism	Cofactors and Vitamins	HMDB00232	1.52	0.1584
Trigonelline (N'-Methylnicotinate)	Nicotinate And Nicotinamide Metabolism	Cofactors and Vitamins	HMDB00875	0.63	0.1010
Pantothenate	Pantothenate And Coa Metabolism	Cofactors and Vitamins	HMDB00210	1.09	0.6267
Flavin Adenine Dinucleotide (Fad)	Riboflavin Metabolism	Cofactors and Vitamins	HMDB01248	1.26	0.0021
Alpha-Cehc Sulfate	Tocopherol Metabolism	Cofactors and Vitamins		2.82	0.1463
Alpha-Tocopherol	Tocopherol Metabolism	Cofactors and Vitamins	HMDB01893	1.00	0.9610
Gamma-Cehc	Tocopherol Metabolism	Cofactors and Vitamins	HMDB01931	1.07	0.7376
Gamma-Cehc Glucuronide*	Tocopherol Metabolism	Cofactors and Vitamins		1.34	0.3618
Gamma-Tocopherol/Beta-Tocopherol	Tocopherol Metabolism	Cofactors and Vitamins		0.82	0.0589
Beta-Cryptoxanthin	Vitamin A Metabolism	Cofactors and Vitamins	HMDB33844	0.57	0.1654
Carotene Diol (1)	Vitamin A Metabolism	Cofactors and Vitamins		0.87	0.4727

Carotene Diol (2)	Vitamin A Metabolism	Cofactors and Vitamins		0.84	0.3832
Carotene Diol (3)	Vitamin A Metabolism	Cofactors and Vitamins		0.76	0.2410
Retinol (Vitamin A)	Vitamin A Metabolism	Cofactors and Vitamins	HMDB00305	1.08	0.4377
Pyridoxal	Vitamin B6 Metabolism	Cofactors and Vitamins	HMDB01545	1.09	0.3157
Pyridoxate	Vitamin B6 Metabolism	Cofactors and Vitamins	HMDB00017	1.72	0.3376
Phosphate	Oxidative Phosphorylation	Energy	HMDB01429	0.93	0.1654
Aconitate [Cis Or Trans]	Tca Cycle	Energy		0.93	0.3311
Alpha-Ketoglutarate	Tca Cycle	Energy	HMDB00208	0.89	0.7205
Citraconate/Glutaconate	Tca Cycle	Energy		0.58	0.0589
Citrate	Tca Cycle	Energy	HMDB00094	1.04	0.3520
Malate	Tca Cycle	Energy	HMDB00156	0.90	0.4377
Succinate	Tca Cycle	Energy	HMDB00254	0.80	0.0078
Succinylcarnitine (C4-Dc)	Tca Cycle	Energy	HMDB61717	1.17	0.2721
11-Ketoetiocholanolone Glucuronide	Androgenic Steroids	Lipid		2.60	0.0570
16A-Hydroxy Dhea 3-Sulfate	Androgenic Steroids	Lipid		1.09	0.8090
5Alpha-Androstan-3Alpha,17Alpha-Diol Monosulfate	Androgenic Steroids	Lipid		1.10	0.8031
5Alpha-Androstan-3Alpha,17Beta-Diol Disulfate	Androgenic Steroids	Lipid		1.77	0.5465
5Alpha-Androstan-3Alpha,17Beta-Diol Monosulfate (1)	Androgenic Steroids	Lipid		0.87	0.6761
5Alpha-Androstan-3Alpha,17Beta-Diol Monosulfate (2)	Androgenic Steroids	Lipid		Excluded	Excluded
5Alpha-Androstan-3Beta,17Alpha-Diol Disulfate	Androgenic Steroids	Lipid		1.18	0.7375
5Alpha-Androstan-3Beta,17Beta-Diol Disulfate	Androgenic Steroids	Lipid	HMDB00493	1.01	0.9745
5Alpha-Androstan-3Beta,17Beta-Diol Monosulfate (2)	Androgenic Steroids	Lipid		0.94	0.8546
Andro Steroid Monosulfate C19H28O6S (1)*	Androgenic Steroids	Lipid	HMDB02759	1.10	0.7376
Androstenediol (3Alpha, 17Alpha) Monosulfate (2)	Androgenic Steroids	Lipid		0.83	0.3646
Androstenediol (3Alpha, 17Alpha) Monosulfate (3)	Androgenic Steroids	Lipid		1.08	0.7601

Androstenediol (3Beta,17Beta) Disulfate (1)	Androgenic Steroids	Lipid	HMDB03818	1.00	0.9984
Androstenediol (3Beta,17Beta) Disulfate (2)	Androgenic Steroids	Lipid	HMDB03818	0.97	0.8808
Androstenediol (3Beta,17Beta) Monosulfate (1)	Androgenic Steroids	Lipid	HMDB03818	0.99	0.9603
Androstenediol (3Beta,17Beta) Monosulfate (2)	Androgenic Steroids	Lipid		0.87	0.6465
Androsterone Sulfate	Androgenic Steroids	Lipid	HMDB02759	1.01	0.9743
Dehydroisoandrosterone Sulfate (Dhea-S)	Androgenic Steroids	Lipid	HMDB01032	0.96	0.8689
Epiandrosterone Sulfate	Androgenic Steroids	Lipid		1.05	0.8776
Etiocholanolone Glucuronide	Androgenic Steroids	Lipid	HMDB04484	1.41	0.3618
Carnitine	Carnitine Metabolism	Lipid	HMDB00062	0.96	0.3832
Deoxycarnitine	Carnitine Metabolism	Lipid	HMDB01161	1.03	0.7254
Ceramide (D16:1/24:1, D18:1/22:1)*	Ceramides	Lipid		0.98	0.9408
Ceramide (D18:1/14:0, D16:1/16:0)*	Ceramides	Lipid		1.02	0.8731
Ceramide (D18:1/20:0, D16:1/22:0, D20:1/18:0)*	Ceramides	Lipid		0.90	0.7438
Ceramide (D18:2/24:1, D18:1/24:2)*	Ceramides	Lipid		1.13	0.2958
Glycosyl Ceramide (D16:1/24:1, D18:1/22:1)*	Ceramides	Lipid		1.08	0.8436
Glycosyl Ceramide (D18:1/20:0, D16:1/22:0)*	Ceramides	Lipid		1.05	0.6966
Glycosyl Ceramide (D18:1/23:1, D17:1/24:1)*	Ceramides	Lipid		1.16	0.7047
Glycosyl Ceramide (D18:2/24:1, D18:1/24:2)*	Ceramides	Lipid		1.17	0.3150
Glycosyl-N-(2-Hydroxynervonoyl)-Sphingosine (D18:1/24:1(2Oh))*	Ceramides	Lipid		0.93	0.7135
Glycosyl-N-Behenoyl-Sphingadienine (D18:2/22:0)*	Ceramides	Lipid		1.03	0.8696
Glycosyl-N-Palmitoyl-Sphingosine (D18:1/16:0)	Ceramides	Lipid		1.11	0.3106
Glycosyl-N-Stearoyl-Sphingosine (D18:1/18:0)	Ceramides	Lipid		1.16	0.3441

Lactosyl-N-Nervonoyl-Sphingosine (D18:1/24:1)*	Ceramides	Lipid		1.12	0.3157
Lactosyl-N-Palmitoyl-Sphingosine (D18:1/16:0)	Ceramides	Lipid		1.11	0.2958
N-Palmitoyl-Sphingosine (D18:1/16:0)	Ceramides	Lipid	HMDB04949	1.19	0.0200
N-Stearoyl-Sphingosine (D18:1/18:0)*	Ceramides	Lipid	HMDB04950	1.28	0.0837
Corticosterone	Corticosteroids	Lipid	HMDB01547	1.20	0.7025
Cortisol	Corticosteroids	Lipid	HMDB00063	1.09	0.4686
Cortisone	Corticosteroids	Lipid	HMDB02802	0.97	0.8349
Linoleoyl-Arachidonoyl-Glycerol (18:2/20:4) [1]*	Diacylglycerol	Lipid	HMDB07257	1.14	0.6586
Linoleoyl-Arachidonoyl-Glycerol (18:2/20:4) [2]*	Diacylglycerol	Lipid	HMDB07257	1.26	0.4727
Linoleoyl-Linoleoyl-Glycerol (18:2/18:2) [1]*	Diacylglycerol	Lipid	HMDB07248	1.09	0.7207
Oleoyl-Arachidonoyl-Glycerol (18:1/20:4) [2]*	Diacylglycerol	Lipid	HMDB07228	1.36	0.4395
[1]	Diacylglycerol	Lipid	HMDB07219	1.16	0.4425
[2]	Diacylglycerol	Lipid	HMDB07219	1.24	0.3109
Oleoyl-Oleoyl-Glycerol (18:1/18:1) [2]*	Diacylglycerol	Lipid	HMDB07218	1.53	0.3193
Palmitoleoyl-Linoleoyl-Glycerol (16:1/18:2) [1]*	Diacylglycerol	Lipid	HMDB07132	1.25	0.3308
Linoleoyl Ethanolamide	Endocannabinoid	Lipid	HMDB12252	0.89	0.5256
N-Oleoylserine	Endocannabinoid	Lipid		0.91	0.3784
N-Oleoyltaurine	Endocannabinoid	Lipid		0.97	0.8734
N-Palmitoylserine	Endocannabinoid	Lipid		1.16	0.4727
N-Palmitoyltaurine	Endocannabinoid	Lipid		1.42	0.1859
N-Stearoyltaurine	Endocannabinoid	Lipid		1.06	0.8090
Oleoyl Ethanolamide	Endocannabinoid	Lipid	HMDB02088	0.91	0.5715
Arachidonoylcholine	Fatty Acid Metabolism (Acyl Choline)	Lipid		1.09	0.7779
Dihomo-Linolenoyl-Choline	Fatty Acid Metabolism (Acyl Choline)	Lipid		1.32	0.4368
Docosahexaenoylcholine	Fatty Acid Metabolism (Acyl Choline)	Lipid		0.95	0.8731
Eicosapentaenoylcholine	Fatty Acid Metabolism (Acyl Choline)	Lipid		0.90	0.8090
Linoleoylcholine*	Fatty Acid Metabolism (Acyl Choline)	Lipid		1.15	0.6448
Oleoylcholine	Fatty Acid Metabolism (Acyl Choline)	Lipid		1.35	0.3494

Palmitoylcholine	Fatty Acid Metabolism (Acyl Choline)	Lipid		1.18	0.5322
Stearoylcholine*	Fatty Acid Metabolism (Acyl Choline)	Lipid		1.21	0.5465
Hexanoylglutamine	Fatty Acid Metabolism (Acyl Glutamine)	Lipid		1.15	0.6877
Butyrylcarnitine (C4)	Fatty Acid Metabolism (Also Bcaa Metabolism)	Lipid	HMDB02013	0.88	0.6076
Methylmalonate (Mma)	Fatty Acid Metabolism (Also Bcaa Metabolism)	Lipid	HMDB00202	1.18	0.6001
Propionylcarnitine (C3)	Fatty Acid Metabolism (Also Bcaa Metabolism)	Lipid	HMDB00824	1.08	0.5322
Propionylglycine	Fatty Acid Metabolism (Also Bcaa Metabolism)	Lipid	HMDB00783	1.00	0.9921
3-Hydroxybutyrylcarnitine (1)	Fatty Acid Metabolism(Acyl Carnitine)	Lipid	HMDB13127	0.97	0.9490
3-Hydroxybutyrylcarnitine (2)	Fatty Acid Metabolism(Acyl Carnitine)	Lipid	HMDB13127	1.14	0.6465
Acetylcarnitine (C2)	Fatty Acid Metabolism(Acyl Carnitine)	Lipid	HMDB00201	0.94	0.5371
Adipoylcarnitine (C6-Dc)	Fatty Acid Metabolism(Acyl Carnitine)	Lipid	HMDB61677	1.52	0.0725
Arachidonoylcarnitine (C20:4)	Fatty Acid Metabolism(Acyl Carnitine)	Lipid		0.80	0.3350
Arachidoylcarnitine (C20)*	Fatty Acid Metabolism(Acyl Carnitine)	Lipid	HMDB06460	1.01	0.9699
Cerotoylcarnitine (C26)*	Fatty Acid Metabolism(Acyl Carnitine)	Lipid	HMDB06347	0.97	0.8296
Cis-4-Decenoylcarnitine (C10:1)	Fatty Acid Metabolism(Acyl Carnitine)	Lipid		1.02	0.9277
Decanoylcarnitine (C10)	Fatty Acid Metabolism(Acyl Carnitine)	Lipid	HMDB00651	1.27	0.6321
Dihomo-Linolenoylcarnitine (20:3N3 Or 6)*	Fatty Acid Metabolism(Acyl Carnitine)	Lipid		0.82	0.3848
Dihomo-Linoleoylcarnitine (C20:2)*	Fatty Acid Metabolism(Acyl Carnitine)	Lipid		0.87	0.4573
Eicosenoylcarnitine (C20:1)*	Fatty Acid Metabolism(Acyl Carnitine)	Lipid		0.91	0.5554
Hexanoylcarnitine (C6)	Fatty Acid Metabolism(Acyl Carnitine)	Lipid	HMDB00705	1.14	0.7617
Laurylcarnitine (C12)	Fatty Acid Metabolism(Acyl Carnitine)	Lipid	HMDB02250	1.20	0.6502
Lignoceroylcarnitine (C24)*	Fatty Acid Metabolism(Acyl Carnitine)	Lipid		0.88	0.1842
Linolenoylcarnitine (C18:3)*	Fatty Acid Metabolism(Acyl Carnitine)	Lipid		0.76	0.1657
Linoleoylcarnitine (C18:2)*	Fatty Acid Metabolism(Acyl Carnitine)	Lipid	HMDB06469	0.80	0.1081
Margaroylcarnitine*	Fatty Acid Metabolism(Acyl Carnitine)	Lipid	HMDB06210	0.89	0.4686
Myristoleoylcarnitine (C14:1)*	Fatty Acid Metabolism(Acyl Carnitine)	Lipid		1.17	0.6372
Myristoylcarnitine (C14)	Fatty Acid Metabolism(Acyl Carnitine)	Lipid	HMDB05066	1.05	0.8540
Nervonoylcarnitine (C24:1)*	Fatty Acid Metabolism(Acyl Carnitine)	Lipid		0.91	0.4686
Octanoylcarnitine (C8)	Fatty Acid Metabolism(Acyl Carnitine)	Lipid	HMDB00791	1.22	0.6586

Oleoylcarnitine (C18:1)	Fatty Acid Metabolism(Acyl Carnitine)	Lipid	HMDB05065	0.87	0.2577
Palmitoleoylcarnitine (C16:1)*	Fatty Acid Metabolism(Acyl Carnitine)	Lipid		1.02	0.9286
Palmitoylcarnitine (C16)	Fatty Acid Metabolism(Acyl Carnitine)	Lipid	HMDB00222	0.95	0.5779
Pimeloylcarnitine/3-Methyladipoylcarnitine (C7-Dc)	Fatty Acid Metabolism(Acyl Carnitine)	Lipid		1.14	0.5322
Stearoylcarnitine (C18)	Fatty Acid Metabolism(Acyl Carnitine)	Lipid	HMDB00848	0.94	0.5779
Suberoylcarnitine (C8-Dc)	Fatty Acid Metabolism(Acyl Carnitine)	Lipid		1.45	0.2741
Ximenoylcarnitine (C26:1)*	Fatty Acid Metabolism(Acyl Carnitine)	Lipid		0.99	0.9286
Hexanoylglycine	Fatty Acid Metabolism(Acyl Glycine)	Lipid	HMDB00701	0.88	0.6465
N-Palmitoylglycine	Fatty Acid Metabolism(Acyl Glycine)	Lipid	HMDB13034	0.97	0.8143
Malonate	Fatty Acid Synthesis	Lipid	HMDB00691	0.81	0.1693
Linoleamide (18:2N6)	Fatty Acid, Amide	Lipid		0.82	0.0626
Oleamide	Fatty Acid, Amide	Lipid	HMDB02117	0.81	0.0204
Palmitic Amide	Fatty Acid, Amide	Lipid	HMDB12273	0.79	0.0203
Stearamide	Fatty Acid, Amide	Lipid	HMDB34146	0.73	0.0031
2-Aminoheptanoate	Fatty Acid, Amino	Lipid		1.26	0.3520
2-Aminooctanoate	Fatty Acid, Amino	Lipid	HMDB00991	0.86	0.3832
13-Methylmyristate (I15:0)	Fatty Acid, Branched	Lipid		0.74	0.0732
15-Methylpalmitate (I17:0)	Fatty Acid, Branched	Lipid		0.79	0.1673
17-Methylstearate (I19:0)	Fatty Acid, Branched	Lipid	HMDB37397	0.85	0.3222
Pristanate	Fatty Acid, Branched	Lipid	HMDB00795	0.67	0.0010
2-Hydroxyglutarate	Fatty Acid, Dicarboxylate	Lipid	HMDB00606	0.97	0.8540
3-Carboxy-4-Methyl-5-Propyl-2-Furanpropanoate (CMPF)	Fatty Acid, Dicarboxylate	Lipid	HMDB61112	0.89	0.7392
Adipate	Fatty Acid, Dicarboxylate	Lipid	HMDB00448	0.99	0.9394
Azelate (Nonanedioate)	Fatty Acid, Dicarboxylate	Lipid	HMDB00784	1.55	0.0489
Docosadioate	Fatty Acid, Dicarboxylate	Lipid		0.90	0.6271
Dodecanedioate	Fatty Acid, Dicarboxylate	Lipid	HMDB00623	1.42	0.2205
Eicosanodioate	Fatty Acid, Dicarboxylate	Lipid		0.83	0.1842
Glutarate (Pentanedioate)	Fatty Acid, Dicarboxylate	Lipid	HMDB00661	1.05	0.8689
Hexadecanedioate	Fatty Acid, Dicarboxylate	Lipid	HMDB00672	1.10	0.6586
Maleate	Fatty Acid, Dicarboxylate	Lipid	HMDB00176	0.55	0.0000
Octadecanedioate	Fatty Acid, Dicarboxylate	Lipid	HMDB00782	1.05	0.8218
Sebacate (Decanedioate)	Fatty Acid, Dicarboxylate	Lipid	HMDB00792	1.03	0.9149

Suberate (Octanedioate)	Fatty Acid, Dicarboxylate	Lipid	HMDB00893	1.26	0.3308
Tetradecanedioate	Fatty Acid, Dicarboxylate	Lipid	HMDB00872	1.14	0.6448
Undecanedioate	Fatty Acid, Dicarboxylate	Lipid	HMDB00888	1.34	0.1742
12,13-Dihome	Fatty Acid, Dihydroxy	Lipid	HMDB04705	0.74	0.2905
9,10-Dihome	Fatty Acid, Dihydroxy	Lipid	HMDB04704	0.85	0.5779
13-Hode + 9-Hode	Fatty Acid, Monohydroxy	Lipid		0.72	0.0303
16-Hydroxypalmitate	Fatty Acid, Monohydroxy	Lipid	HMDB06294	0.89	0.3834
2-Hydroxydecanoate	Fatty Acid, Monohydroxy	Lipid		0.79	0.1988
2-Hydroxylaurate	Fatty Acid, Monohydroxy	Lipid		0.92	0.5371
2-Hydroxynervonate*	Fatty Acid, Monohydroxy	Lipid		0.98	0.8457
2-Hydroxyoctanoate	Fatty Acid, Monohydroxy	Lipid	HMDB02264	0.89	0.4266
2-Hydroxypalmitate	Fatty Acid, Monohydroxy	Lipid	HMDB31057	0.91	0.1915
2-Hydroxystearate	Fatty Acid, Monohydroxy	Lipid		0.86	0.0228
3-Hydroxydecanoate	Fatty Acid, Monohydroxy	Lipid	HMDB02203	0.95	0.8578
3-Hydroxyhexanoate	Fatty Acid, Monohydroxy	Lipid		0.94	0.6784
3-Hydroxylaurate	Fatty Acid, Monohydroxy	Lipid	HMDB00387	0.75	0.1187
3-Hydroxyoctanoate	Fatty Acid, Monohydroxy	Lipid	HMDB01954	1.07	0.8419
3-Hydroxysebacate	Fatty Acid, Monohydroxy	Lipid	HMDB00350	1.43	0.3466
5-Hydroxyhexanoate	Fatty Acid, Monohydroxy	Lipid	HMDB00525	1.21	0.4727
9-Hydroxystearate	Fatty Acid, Monohydroxy	Lipid	HMDB61661	0.79	0.3028
Glycerol	Glycerolipid Metabolism	Lipid	HMDB00131	0.92	0.6465
Glycerol 3-Phosphate	Glycerolipid Metabolism	Lipid	HMDB00126	0.67	0.0000
Glycerophosphoglycerol	Glycerolipid Metabolism	Lipid		1.14	0.5624
Chiro-Inositol	Inositol Metabolism	Lipid	HMDB34220	1.03	0.9607
Myo-Inositol	Inositol Metabolism	Lipid	HMDB00211	1.08	0.6821
3-Hydroxybutyrate (Bhba)	Ketone Bodies	Lipid	HMDB00357	0.80	0.6001
Acetoacetate	Ketone Bodies	Lipid	HMDB00060	0.87	0.7025
10-Heptadecenoate (17:1N7)	Long Chain Fatty Acid	Lipid	HMDB60038	0.80	0.3134
10-Nonadecenoate (19:1N9)	Long Chain Fatty Acid	Lipid	HMDB13622	0.82	0.3157
Arachidate (20:0)	Long Chain Fatty Acid	Lipid	HMDB02212	0.94	0.4425
Eicosenoate (20:1)	Long Chain Fatty Acid	Lipid	HMDB02231	0.83	0.3822
Erucate (22:1N9)	Long Chain Fatty Acid	Lipid	HMDB02068	1.06	0.8419
Margarate (17:0)	Long Chain Fatty Acid	Lipid	HMDB02259	0.82	0.1986

Myristate (14:0)	Long Chain Fatty Acid	Lipid	HMDB00806	0.76	0.1213
Myristoleate (14:1N5)	Long Chain Fatty Acid	Lipid	HMDB02000	0.80	0.4377
Nonadecanoate (19:0)	Long Chain Fatty Acid	Lipid	HMDB00772	0.88	0.2824
Oleate/Vaccenate (18:1)	Long Chain Fatty Acid	Lipid		0.81	0.2337
Palmitate (16:0)	Long Chain Fatty Acid	Lipid	HMDB00220	0.81	0.1308
Palmitoleate (16:1N7)	Long Chain Fatty Acid	Lipid	HMDB03229	0.77	0.3929
Pentadecanoate (15:0)	Long Chain Fatty Acid	Lipid	HMDB00826	0.82	0.1081
Stearate (18:0)	Long Chain Fatty Acid	Lipid	HMDB00827	0.87	0.1738
1-Arachidonoyl-Gpa (20:4)	Lysophospholipid	Lipid		0.91	0.7392
1-Arachidonoyl-Gpc (20:4N6)*	Lysophospholipid	Lipid	HMDB10395	0.95	0.6905
1-Arachidonoyl-Gpe (20:4N6)*	Lysophospholipid	Lipid	HMDB11517	1.08	0.4039
1-Arachidonoyl-Gpi (20:4)*	Lysophospholipid	Lipid	HMDB61690	0.89	0.3986
1-Lignoceroyl-Gpc (24:0)	Lysophospholipid	Lipid	HMDB10405	0.98	0.8808
1-Linolenoyl-Gpc (18:3)*	Lysophospholipid	Lipid	HMDB10388	0.98	0.8761
1-Linoleoyl-Gpa (18:2)*	Lysophospholipid	Lipid	HMDB07856	0.71	0.0205
1-Linoleoyl-Gpc (18:2)	Lysophospholipid	Lipid	HMDB10386	0.96	0.6905
1-Linoleoyl-Gpe (18:2)*	Lysophospholipid	Lipid	HMDB11507	1.10	0.5343
1-Linoleoyl-Gpg (18:2)*	Lysophospholipid	Lipid		0.95	0.7205
1-Linoleoyl-Gpi (18:2)*	Lysophospholipid	Lipid		0.90	0.5066
1-Oleoyl-Gpc (18:1)	Lysophospholipid	Lipid	HMDB02815	1.01	0.8611
1-Oleoyl-Gpe (18:1)	Lysophospholipid	Lipid	HMDB11506	1.06	0.6905
1-Oleoyl-Gpg (18:1)*	Lysophospholipid	Lipid		1.25	0.3832
1-Oleoyl-Gpi (18:1)*	Lysophospholipid	Lipid		0.92	0.6905
1-Oleoyl-Gps (18:1)	Lysophospholipid	Lipid	HMDB61694	Excluded	Excluded
1-Palmitoleoyl-Gpc (16:1)*	Lysophospholipid	Lipid	HMDB10383	0.99	0.9610
1-Palmitoyl-Gpa (16:0)	Lysophospholipid	Lipid	HMDB00327	0.93	0.6966
1-Palmitoyl-Gpc (16:0)	Lysophospholipid	Lipid	HMDB10382	0.95	0.3929
1-Palmitoyl-Gpe (16:0)	Lysophospholipid	Lipid	HMDB11503	1.03	0.7625
1-Palmitoyl-Gpg (16:0)*	Lysophospholipid	Lipid		1.04	0.8090
1-Palmitoyl-Gpi (16:0)	Lysophospholipid	Lipid	HMDB61695	1.05	0.8540
1-Stearoyl-Gpc (18:0)	Lysophospholipid	Lipid	HMDB10384	0.95	0.5779
1-Stearoyl-Gpe (18:0)	Lysophospholipid	Lipid	HMDB11130	1.09	0.4069
1-Stearoyl-Gpg (18:0)	Lysophospholipid	Lipid		0.97	0.8961

1-Stearoyl-Gpi (18:0)	Lysophospholipid	Lipid	HMDB61696	0.92	0.6915
1-Stearoyl-Gps (18:0)*	Lysophospholipid	Lipid		Excluded	Excluded
2-Palmitoleoyl-Gpc (16:1)*	Lysophospholipid	Lipid	HMDB10383	1.00	0.9921
2-Palmitoyl-Gpc (16:0)*	Lysophospholipid	Lipid	HMDB61702	0.89	0.1882
2-Stearoyl-Gpe (18:0)*	Lysophospholipid	Lipid	HMDB11129	0.96	0.7445
1-(1-Enyl-Oleoyl)-Gpe (P-18:1)*	Lysoplasmalogen	Lipid		1.25	0.1338
1-(1-Enyl-Palmitoyl)-Gpc (P-16:0)*	Lysoplasmalogen	Lipid	HMDB10407	0.91	0.3939
1-(1-Enyl-Palmitoyl)-Gpe (P-16:0)*	Lysoplasmalogen	Lipid		1.30	0.0132
1-(1-Enyl-Stearoyl)-Gpe (P-18:0)*	Lysoplasmalogen	Lipid		1.28	0.0522
10-Undecenoate (11:1N1)	Medium Chain Fatty Acid	Lipid	HMDB33724	0.64	0.0000
5-Dodecenoate (12:1N7)	Medium Chain Fatty Acid	Lipid	HMDB00529	0.91	0.8090
Caprate (10:0)	Medium Chain Fatty Acid	Lipid	HMDB00511	0.85	0.4686
Caproate (6:0)	Medium Chain Fatty Acid	Lipid	HMDB00535	1.37	0.1003
Caprylate (8:0)	Medium Chain Fatty Acid	Lipid	HMDB00482	1.19	0.6448
Heptanoate (7:0)	Medium Chain Fatty Acid	Lipid	HMDB00666	1.86	0.0000
Laurate (12:0)	Medium Chain Fatty Acid	Lipid	HMDB00638	0.92	0.7357
3-Hydroxy-3-Methylglutarate	Mevalonate Metabolism	Lipid	HMDB00355	1.21	0.4727
1-Arachidonylglycerol (20:4)	Monoacylglycerol	Lipid	HMDB11549	0.87	0.6230
1-Dihomo-Linolenylglycerol (20:3)	Monoacylglycerol	Lipid		0.80	0.3441
1-Linolenoylglycerol (18:3)	Monoacylglycerol	Lipid	HMDB11569	0.58	0.0103
1-Linoleoylglycerol (18:2)	Monoacylglycerol	Lipid		0.60	0.0004
1-Myristoylglycerol (14:0)	Monoacylglycerol	Lipid	HMDB11561	0.79	0.3106
1-Oleoylglycerol (18:1)	Monoacylglycerol	Lipid	HMDB11567	0.70	0.0301
1-Palmitoleoylglycerol (16:1)*	Monoacylglycerol	Lipid	HMDB11565	0.48	0.0016
1-Palmitoylglycerol (16:0)	Monoacylglycerol	Lipid	HMDB31074	0.71	0.1584
2-Arachidonoylglycerol (20:4)	Monoacylglycerol	Lipid	HMDB04666	1.18	0.6700
2-Linoleoylglycerol (18:2)	Monoacylglycerol	Lipid	HMDB11538	0.63	0.1273
2-Oleoylglycerol (18:1)	Monoacylglycerol	Lipid	HMDB11537	0.67	0.2777
2-Palmitoylglycerol (16:0)	Monoacylglycerol	Lipid	HMDB11533	1.35	0.4686
1,2-Dilinoleoyl-Gpc (18:2/18:2)	Phosphatidylcholine (Pc)	Lipid	HMDB08138	0.96	0.7176
1,2-Dipalmitoyl-Gpc (16:0/16:0)	Phosphatidylcholine (Pc)	Lipid	HMDB00564	1.06	0.2824
1-Linoleoyl-2-Arachidonoyl-Gpc (18:2/20:4N6)*	Phosphatidylcholine (Pc)	Lipid	HMDB08147	0.89	0.0881

1-Linoleoyl-2-Linolenoyl-Gpc (18:2/18:3)*	Phosphatidylcholine (Pc)	Lipid	HMDB08141	1.03	0.8814
1-Myristoyl-2-Arachidonoyl-Gpc (14:0/20:4)*	Phosphatidylcholine (Pc)	Lipid	HMDB07883	0.91	0.5802
(14:0/16:0)	Phosphatidylcholine (Pc)	Lipid	HMDB07869	1.05	0.7740
1-Oleoyl-2-Docosahexaenoyl-Gpc (18:1/22:6)*	Phosphatidylcholine (Pc)	Lipid	HMDB08123	1.00	0.9533
1-Palmitoleoyl-2-Linolenoyl-Gpc (16:1/18:3)*	Phosphatidylcholine (Pc)	Lipid	HMDB08008	1.04	0.8353
1-Palmitoyl-2-Arachidonoyl-Gpc (16:0/20:4N6)	Phosphatidylcholine (Pc)	Lipid	HMDB07982	0.96	0.4302
1-Palmitoyl-2-Dihomo-Linolenoyl-Gpc (16:0/20:3N3 Or 6)*	Phosphatidylcholine (Pc)	Lipid		1.05	0.2824
1-Palmitoyl-2-Docosahexaenoyl-Gpc (16:0/22:6)	Phosphatidylcholine (Pc)	Lipid	HMDB07991	0.97	0.6465
1-Palmitoyl-2-Gamma-Linolenoyl-Gpc (16:0/18:3N6)*	Phosphatidylcholine (Pc)	Lipid	HMDB07974	1.06	0.7617
(16:0/18:2)	Phosphatidylcholine (Pc)	Lipid	HMDB07973	1.00	0.9178
1-Palmitoyl-2-Oleoyl-Gpc (16:0/18:1)	Phosphatidylcholine (Pc)	Lipid	HMDB07972	1.06	0.1859
1-Palmitoyl-2-Palmitoleoyl-Gpc (16:0/16:1)*	Phosphatidylcholine (Pc)	Lipid	HMDB07969	1.12	0.4375
1-Palmitoyl-2-Stearoyl-Gpc (16:0/18:0)	Phosphatidylcholine (Pc)	Lipid	HMDB07970	1.12	0.0783
1-Stearoyl-2-Arachidonoyl-Gpc (18:0/20:4)	Phosphatidylcholine (Pc)	Lipid	HMDB08048	0.96	0.4639
1-Stearoyl-2-Docosahexaenoyl-Gpc (18:0/22:6)	Phosphatidylcholine (Pc)	Lipid	HMDB08057	0.97	0.8090
(18:0/18:2)*	Phosphatidylcholine (Pc)	Lipid	HMDB08039	0.98	0.6916
1-Stearoyl-2-Oleoyl-Gpc (18:0/18:1)	Phosphatidylcholine (Pc)	Lipid	HMDB08038	1.07	0.3959
1,2-Dilinoleoyl-Gpe (18:2/18:2)*	Phosphatidylethanolamine (Pe)	Lipid	HMDB09093	1.38	0.5899
1-Linoleoyl-2-Arachidonoyl-Gpe (18:2/20:4)*	Phosphatidylethanolamine (Pe)	Lipid	HMDB09102	1.19	0.4105
1-Oleoyl-2-Arachidonoyl-Gpe (18:1/20:4)*	Phosphatidylethanolamine (Pe)	Lipid	HMDB09069	1.43	0.0120
1-Oleoyl-2-Docosahexaenoyl-Gpe (18:1/22:6)*	Phosphatidylethanolamine (Pe)	Lipid		0.98	0.9122
1-Oleoyl-2-Linoleoyl-Gpe (18:1/18:2)*	Phosphatidylethanolamine (Pe)	Lipid	HMDB05349	1.22	0.4377

1-Palmitoyl-2-Arachidonoyl-Gpe (16:0/20:4)*	Phosphatidylethanolamine (Pe)	Lipid	HMDB05323	1.23	0.1490
1-Palmitoyl-2-Docosahexaenoyl-Gpe (16:0/22:6)*	Phosphatidylethanolamine (Pe)	Lipid	HMDB05324	0.99	0.9589
(16:0/18:2)	Phosphatidylethanolamine (Pe)	Lipid	HMDB05322	1.28	0.1684
1-Palmitoyl-2-Oleoyl-Gpe (16:0/18:1)	Phosphatidylethanolamine (Pe)	Lipid	HMDB05320	1.27	0.1842
1-Stearoyl-2-Arachidonoyl-Gpe (18:0/20:4)	Phosphatidylethanolamine (Pe)	Lipid	HMDB09003	1.27	0.0339
1-Stearoyl-2-Docosahexaenoyl-Gpe (18:0/22:6)*	Phosphatidylethanolamine (Pe)	Lipid	HMDB05334	1.01	0.9509
(18:0/18:2)*	Phosphatidylethanolamine (Pe)	Lipid	HMDB08994	1.19	0.3350
1-Stearoyl-2-Oleoyl-Gpe (18:0/18:1)	Phosphatidylethanolamine (Pe)	Lipid	HMDB08993	1.23	0.3098
1-Palmitoyl-2-Arachidonoyl-Gpi (16:0/20:4)*	Phosphatidylinositol (Pi)	Lipid	HMDB09789	1.07	0.6321
(16:0/18:2)	Phosphatidylinositol (Pi)	Lipid	HMDB09784	1.09	0.6267
1-Palmitoyl-2-Oleoyl-Gpi (16:0/18:1)*	Phosphatidylinositol (Pi)	Lipid	HMDB09783	1.08	0.6138
1-Stearoyl-2-Arachidonoyl-Gpi (18:0/20:4)	Phosphatidylinositol (Pi)	Lipid	HMDB09815	1.02	0.8368
1-Stearoyl-2-Linoleoyl-Gpi (18:0/18:2)	Phosphatidylinositol (Pi)	Lipid	HMDB09809	1.00	0.9792
1-Stearoyl-2-Oleoyl-Gpi (18:0/18:1)*	Phosphatidylinositol (Pi)	Lipid		0.94	0.6579
1-Stearoyl-2-Arachidonoyl-Gps (18:0/20:4)	Phosphatidylserine (Ps)	Lipid	HMDB12383	13.81	0.0000
1-Stearoyl-2-Oleoyl-Gps (18:0/18:1)	Phosphatidylserine (Ps)	Lipid	HMDB10163	16.57	0.0000
Choline	Phospholipid Metabolism	Lipid	HMDB00097	1.03	0.6465
Choline Phosphate	Phospholipid Metabolism	Lipid	HMDB01565	1.67	0.0000
Glycerophosphoethanolamine	Phospholipid Metabolism	Lipid	HMDB00114	1.12	0.2057
Glycerophosphoinositol*	Phospholipid Metabolism	Lipid		1.46	0.1213
Glycerophosphorylcholine (Gpc)	Phospholipid Metabolism	Lipid	HMDB00086	0.95	0.7740
Phosphoethanolamine	Phospholipid Metabolism	Lipid	HMDB00224	2.90	0.0000
Trimethylamine N-Oxide	Phospholipid Metabolism	Lipid	HMDB00925	1.34	0.3308
1-(1-Enyl-Palmitoyl)-2-Arachidonoyl-Gpc (P-16:0/20:4)*	Plasmalogen	Lipid	HMDB11220	0.87	0.0571
1-(1-Enyl-Palmitoyl)-2-Arachidonoyl-Gpe (P-16:0/20:4)*	Plasmalogen	Lipid	HMDB11352	1.08	0.4175
1-(1-Enyl-Palmitoyl)-2-Linoleoyl-Gpc (P-16:0/18:2)*	Plasmalogen	Lipid	HMDB11211	0.81	0.0062

1-(1-Enyl-Palmitoyl)-2-Linoleoyl-Gpe (P-16:0/18:2)*	Plasmalogen	Lipid	HMDB11343	0.88	0.2784
1-(1-Enyl-Palmitoyl)-2-Oleoyl-Gpc (P-16:0/18:1)*	Plasmalogen	Lipid		0.94	0.5236
1-(1-Enyl-Palmitoyl)-2-Oleoyl-Gpe (P-16:0/18:1)*	Plasmalogen	Lipid	HMDB11342	1.03	0.8031
1-(1-Enyl-Palmitoyl)-2-Palmitoleoyl-Gpc (P-16:0/16:1)*	Plasmalogen	Lipid	HMDB11207	0.97	0.8031
1-(1-Enyl-Palmitoyl)-2-Palmitoyl-Gpc (P-16:0/16:0)*	Plasmalogen	Lipid	HMDB11206	0.96	0.6328
1-(1-Enyl-Stearoyl)-2-Arachidonoyl-Gpe (P-18:0/20:4)*	Plasmalogen	Lipid	HMDB05779	1.06	0.6388
1-(1-Enyl-Stearoyl)-2-Linoleoyl-Gpe (P-18:0/18:2)*	Plasmalogen	Lipid	HMDB11376	0.95	0.7207
1-(1-Enyl-Stearoyl)-2-Oleoyl-Gpe (P-18:0/18:1)	Plasmalogen	Lipid	HMDB11375	1.05	0.7625
Adrenate (22:4N6)	Polyunsaturated Fatty Acid (N3 And N6)	Lipid	HMDB02226	0.87	0.5021
Arachidonate (20:4N6)	Polyunsaturated Fatty Acid (N3 And N6)	Lipid	HMDB01043	0.87	0.4175
Dihomo-Linoleate (20:2N6)	Polyunsaturated Fatty Acid (N3 And N6)	Lipid	HMDB05060	0.83	0.3375
Dihomo-Linolenate (20:3N3 Or N6)	Polyunsaturated Fatty Acid (N3 And N6)	Lipid	HMDB02925	0.86	0.3520
Docosadienoate (22:2N6)	Polyunsaturated Fatty Acid (N3 And N6)	Lipid	HMDB61714	0.93	0.6859
Docosahexaenoate (Dha; 22:6N3)	Polyunsaturated Fatty Acid (N3 And N6)	Lipid	HMDB02183	0.70	0.0303
Docosapentaenoate (N3 Dpa; 22:5N3)	Polyunsaturated Fatty Acid (N3 And N6)	Lipid	HMDB06528	0.79	0.2826
Docosapentaenoate (N6 Dpa; 22:5N6)	Polyunsaturated Fatty Acid (N3 And N6)	Lipid	HMDB01976	0.82	0.3123
Docosatrienoate (22:3N3)	Polyunsaturated Fatty Acid (N3 And N6)	Lipid	HMDB02823	0.91	0.6905
Docosatrienoate (22:3N6)*	Polyunsaturated Fatty Acid (N3 And N6)	Lipid		0.80	0.5371
Eicosapentaenoate (Epa; 20:5N3)	Polyunsaturated Fatty Acid (N3 And N6)	Lipid	HMDB01999	0.69	0.0354
Hexadecadienoate (16:2N6)	Polyunsaturated Fatty Acid (N3 And N6)	Lipid	HMDB00477	0.77	0.2824
Linoleate (18:2N6)	Polyunsaturated Fatty Acid (N3 And N6)	Lipid	HMDB00673	0.76	0.0725
Linolenate [Alpha Or Gamma; (18:3N3 Or 6)]	Polyunsaturated Fatty Acid (N3 And N6)	Lipid	HMDB03073	0.76	0.1703
Nisinate (24:6N3)	Polyunsaturated Fatty Acid (N3 And N6)	Lipid	HMDB02007	0.80	0.5431
Stearidonate (18:4N3)	Polyunsaturated Fatty Acid (N3 And N6)	Lipid	HMDB06547	0.89	0.6761
17Alpha-Hydroxypregnanolone Glucuronide	Pregnenolone Steroids	Lipid		Excluded	Excluded
Sulfate	Pregnenolone Steroids	Lipid	HMDB00416	1.23	0.6001

21-Hydroxypregnenolone Disulfate	Pregnenolone Steroids	Lipid			1.19	0.4639
21-Hydroxypregnenolone Monosulfate (1)	Pregnenolone Steroids	Lipid		Excluded	Excluded	
Pregnenolone Sulfate	Pregnenolone Steroids	Lipid	HMDB00774		1.11	0.7183
Chenodeoxycholate	Primary Bile Acid Metabolism	Lipid	HMDB00518		0.87	0.8334
Cholate	Primary Bile Acid Metabolism	Lipid	HMDB00619		0.70	0.6905
Glycochenodeoxycholate	Primary Bile Acid Metabolism	Lipid	HMDB00637		1.21	0.6905
Glycochenodeoxycholate Glucuronide (1)	Primary Bile Acid Metabolism	Lipid			1.31	0.4164
Glycochenodeoxycholate Sulfate	Primary Bile Acid Metabolism	Lipid			1.13	0.6939
Glycocholate	Primary Bile Acid Metabolism	Lipid	HMDB00138		1.04	0.9408
Tauro-Beta-Muricholate	Primary Bile Acid Metabolism	Lipid	HMDB00932		1.04	0.9603
Taurochenodeoxycholate	Primary Bile Acid Metabolism	Lipid	HMDB00951		1.17	0.8353
Taurocholate	Primary Bile Acid Metabolism	Lipid	HMDB00036		1.02	0.9832
5Alpha-Pregnan-3(Alpha Or Beta),20Beta-Diol Disulfate	Progestin Steroids	Lipid			2.20	0.1654
5Alpha-Pregnan-3Beta,20Alpha-Diol Disulfate	Progestin Steroids	Lipid			1.42	0.3387
5Alpha-Pregnan-3Beta,20Alpha-Diol Monosulfate (2)	Progestin Steroids	Lipid			1.00	0.9921
5Alpha-Pregnan-3Beta,20Beta-Diol Monosulfate (1)	Progestin Steroids	Lipid			1.17	0.6305
C21H34O5S*	Progestin Steroids	Lipid			1.04	0.8814
Pregnanediol-3-Glucuronide	Progestin Steroids	Lipid	HMDB10318		1.26	0.4696
Sulfate	Progestin Steroids	Lipid			1.49	0.3226
Pregnen-Diol Disulfate C21H34O8S2*	Progestin Steroids	Lipid			1.32	0.3832
3B-Hydroxy-5-Cholenoic Acid	Secondary Bile Acid Metabolism	Lipid	HMDB00308		1.23	0.6586
7-Ketodeoxycholate	Secondary Bile Acid Metabolism	Lipid	HMDB00391	Excluded	Excluded	
Deoxycholate	Secondary Bile Acid Metabolism	Lipid	HMDB00626		1.18	0.7119
Glycocholenate Sulfate*	Secondary Bile Acid Metabolism	Lipid			1.16	0.4727
Glycodeoxycholate	Secondary Bile Acid Metabolism	Lipid	HMDB00631		1.33	0.4348
Glycodeoxycholate Glucuronide (1)	Secondary Bile Acid Metabolism	Lipid			1.46	0.3832
Glycodeoxycholate Sulfate	Secondary Bile Acid Metabolism	Lipid			1.25	0.4727
Glycohyocholate	Secondary Bile Acid Metabolism	Lipid			0.96	0.9173
Glycolithocholate	Secondary Bile Acid Metabolism	Lipid	HMDB00698		1.62	0.2173

Glycolithocholate Sulfate*	Secondary Bile Acid Metabolism	Lipid	HMDB02639	1.27	0.4377
Glycoursodeoxycholate	Secondary Bile Acid Metabolism	Lipid	HMDB00708	1.32	0.5624
Hyocholate	Secondary Bile Acid Metabolism	Lipid	HMDB00760	0.90	0.8656
Isoursodeoxycholate	Secondary Bile Acid Metabolism	Lipid	HMDB00686	1.22	0.6899
Taurocholenate Sulfate	Secondary Bile Acid Metabolism	Lipid		1.04	0.8689
Taurodeoxycholate	Secondary Bile Acid Metabolism	Lipid	HMDB00896	1.30	0.6341
Taurolithocholate 3-Sulfate	Secondary Bile Acid Metabolism	Lipid	HMDB02580	1.01	0.9832
Tauroursodeoxycholate	Secondary Bile Acid Metabolism	Lipid	HMDB00874	1.53	0.6415
Ursodeoxycholate	Secondary Bile Acid Metabolism	Lipid	HMDB00946	1.19	0.8141
Ursodeoxycholate Sulfate (1)	Secondary Bile Acid Metabolism	Lipid		1.30	0.5002
Behenoyl Dihydrosphingomyelin (D18:0/22:0)*	Sphingolipid Metabolism	Lipid	HMDB12091	0.96	0.7357
(D18:1/22:0)*	Sphingolipid Metabolism	Lipid	HMDB12103	1.00	0.9510
Lactosyl-N-Behenoyl-Sphingosine (D18:1/22:0)*	Sphingolipid Metabolism	Lipid		1.58	0.0032
(D18:1/24:0)	Sphingolipid Metabolism	Lipid		0.96	0.6546
Myristoyl Dihydrosphingomyelin (D18:0/14:0)*	Sphingolipid Metabolism	Lipid	HMDB12085	0.96	0.6700
N-Behenoyl-Sphingadienine (D18:2/22:0)*	Sphingolipid Metabolism	Lipid		0.92	0.8031
N-Palmitoyl-Heptadecasphingosine (D17:1/16:0)*	Sphingolipid Metabolism	Lipid		1.19	0.4397
N-Palmitoyl-Sphingadienine (D18:2/16:0)*	Sphingolipid Metabolism	Lipid		1.09	0.4172
N-Palmitoyl-Sphinganine (D18:0/16:0)	Sphingolipid Metabolism	Lipid	HMDB11760	1.13	0.4727
Palmitoyl Dihydrosphingomyelin (D18:0/16:0)*	Sphingolipid Metabolism	Lipid		0.99	0.8854
Palmitoyl Sphingomyelin (D18:1/16:0)	Sphingolipid Metabolism	Lipid		1.04	0.3832
Sphinganine	Sphingolipid Metabolism	Lipid	HMDB00269	1.76	0.0043
Sphinganine-1-Phosphate	Sphingolipid Metabolism	Lipid	HMDB01383	1.08	0.6446
Sphingomyelin (D17:1/16:0, D18:1/15:0, D16:1/17:0)*	Sphingolipid Metabolism	Lipid		0.99	0.8961
Sphingomyelin (D17:2/16:0, D18:2/15:0)*	Sphingolipid Metabolism	Lipid		0.90	0.4417
Sphingomyelin (D18:0/18:0, D19:0/17:0)*	Sphingolipid Metabolism	Lipid	HMDB12087	1.04	0.8378

Sphingomyelin (D18:0/20:0, D16:0/22:0)*	Sphingolipid Metabolism	Lipid		0.96	0.8236
Sphingomyelin (D18:1/14:0, D16:1/16:0)*	Sphingolipid Metabolism	Lipid	HMDB12097	0.98	0.8218
Sphingomyelin (D18:1/17:0, D17:1/18:0, D19:1/16:0)	Sphingolipid Metabolism	Lipid		0.99	0.9069
D18:2/18:0)	Sphingolipid Metabolism	Lipid	HMDB12101	1.01	0.9280
Sphingomyelin (D18:1/19:0, D19:1/18:0)*	Sphingolipid Metabolism	Lipid		0.96	0.7489
Sphingomyelin (D18:1/20:0, D16:1/22:0)*	Sphingolipid Metabolism	Lipid	HMDB12102	0.97	0.6267
Sphingomyelin (D18:1/20:1, D18:2/20:0)*	Sphingolipid Metabolism	Lipid		0.99	0.8540
Sphingomyelin (D18:1/20:2, D18:2/20:1, D16:1/22:2)*	Sphingolipid Metabolism	Lipid		1.01	0.8996
Sphingomyelin (D18:1/21:0, D17:1/22:0, D16:1/23:0)*	Sphingolipid Metabolism	Lipid		0.88	0.1909
Sphingomyelin (D18:1/22:1, D18:2/22:0, D16:1/24:1)*	Sphingolipid Metabolism	Lipid	HMDB12104	0.96	0.4175
Sphingomyelin (D18:1/22:2, D18:2/22:1, D16:1/24:2)*	Sphingolipid Metabolism	Lipid		1.01	0.9398
Sphingomyelin (D18:1/24:1, D18:2/24:0)*	Sphingolipid Metabolism	Lipid	HMDB12107	1.02	0.8142
Sphingomyelin (D18:1/25:0, D19:0/24:1, D20:1/23:0, D19:1/24:0)*	Sphingolipid Metabolism	Lipid		0.90	0.3573
Sphingomyelin (D18:2/14:0, D18:1/14:1)*	Sphingolipid Metabolism	Lipid		0.94	0.6601
Sphingomyelin (D18:2/16:0, D18:1/16:1)*	Sphingolipid Metabolism	Lipid		1.05	0.4972
Sphingomyelin (D18:2/18:1)*	Sphingolipid Metabolism	Lipid		1.00	0.9741
Sphingomyelin (D18:2/21:0, D16:2/23:0)*	Sphingolipid Metabolism	Lipid		0.92	0.4481
Sphingomyelin (D18:2/23:0, D18:1/23:1, D17:1/24:1)*	Sphingolipid Metabolism	Lipid		0.92	0.3832
Sphingomyelin (D18:2/23:1)*	Sphingolipid Metabolism	Lipid		0.95	0.6859
Sphingomyelin (D18:2/24:1, D18:1/24:2)*	Sphingolipid Metabolism	Lipid		1.03	0.7205

Sphingomyelin (D18:2/24:2)*	Sphingolipid Metabolism	Lipid		1.07	0.5256
Sphingosine	Sphingolipid Metabolism	Lipid	HMDB00252	1.57	0.0032
Sphingosine 1-Phosphate	Sphingolipid Metabolism	Lipid	HMDB00277	1.08	0.3848
Stearoyl Sphingomyelin (D18:1/18:0)	Sphingolipid Metabolism	Lipid	HMDB01348	1.02	0.8589
Tricosanoyl Sphingomyelin (D18:1/23:0)*	Sphingolipid Metabolism	Lipid	HMDB12105	0.89	0.1307
3Beta,7Alpha-Dihydroxy-5-Cholestenoate	Sterol	Lipid		0.89	0.2334
Cholestenoate	Sterol	Lipid		1.11	0.3441
3Beta-Hydroxy-5-Cholestenoate	Sterol	Lipid		0.92	0.4727
7-Alpha-Hydroxy-3-Oxo-4-Cholestenoate (7-Hoca)	Sterol	Lipid	HMDB12458	0.87	0.1081
Cholesterol	Sterol	Lipid	HMDB00067	1.08	0.4302
Allantoin	Purine Metabolism, (Hypo)Xanthine/Inosine Containing	Nucleotide	HMDB00462	1.12	0.1010
Hypoxanthine	Purine Metabolism, (Hypo)Xanthine/Inosine Containing	Nucleotide	HMDB00157	1.37	0.3123
Inosine	Purine Metabolism, (Hypo)Xanthine/Inosine Containing	Nucleotide	HMDB00195	4.61	0.0834
N1-Methylinosine	Purine Metabolism, (Hypo)Xanthine/Inosine Containing	Nucleotide	HMDB02721	1.51	0.0032
Urate	Purine Metabolism, (Hypo)Xanthine/Inosine Containing	Nucleotide	HMDB00289	1.06	0.4161
Xanthine	Purine Metabolism, (Hypo)Xanthine/Inosine Containing	Nucleotide	HMDB00292	0.67	0.3832
Xanthosine	Purine Metabolism, (Hypo)Xanthine/Inosine Containing	Nucleotide	HMDB00299	Excluded	Excluded
Adenine	Purine Metabolism, Adenine Containing	Nucleotide	HMDB00034	1.12	0.7025
Adenosine	Purine Metabolism, Adenine Containing	Nucleotide	HMDB00050	1.74	0.0192
Adenosine 3',5'-Cyclic Monophosphate (Camp)	Purine Metabolism, Adenine Containing	Nucleotide	HMDB00058	1.11	0.4727
Adenosine 5'-Monophosphate (Amp)	Purine Metabolism, Adenine Containing	Nucleotide	HMDB00045	3.06	0.0132
N1-Methyladenosine	Purine Metabolism, Adenine Containing	Nucleotide	HMDB03331	1.10	0.0544
N6-Carbamoylthreonyladenosine	Purine Metabolism, Adenine Containing	Nucleotide	HMDB41623	1.20	0.0800
N6-Succinyladenosine	Purine Metabolism, Adenine Containing	Nucleotide	HMDB00912	1.15	0.6001
7-Methylguanine	Purine Metabolism, Guanine Containing	Nucleotide	HMDB00897	1.04	0.5779

N2,N2-Dimethylguanosine	Purine Metabolism, Guanine Containing	Nucleotide	HMDB04824	1.22	0.0360
2'-O-Methylcytidine	Pyrimidine Metabolism, Cytidine Containing	Nucleotide		1.01	0.9509
3-Methylcytidine	Pyrimidine Metabolism, Cytidine Containing	Nucleotide		1.08	0.6740
Cytidine	Pyrimidine Metabolism, Cytidine Containing	Nucleotide	HMDB00089	1.92	0.2824
Cytosine	Pyrimidine Metabolism, Cytidine Containing	Nucleotide	HMDB00630	1.69	0.2264
N4-Acetylcytidine	Pyrimidine Metabolism, Cytidine Containing	Nucleotide	HMDB05923	1.31	0.0755
Dihydroorotate	Pyrimidine Metabolism, Orotate Containing	Nucleotide	HMDB03349	0.56	0.0029
Orotate	Pyrimidine Metabolism, Orotate Containing	Nucleotide	HMDB00226	0.63	0.4302
Orotidine	Pyrimidine Metabolism, Orotate Containing	Nucleotide	HMDB00788	0.57	0.6565
3-Aminoisobutyrate	Pyrimidine Metabolism, Thymine Containing	Nucleotide	HMDB03911	1.13	0.5066
5,6-Dihydrothymine	Pyrimidine Metabolism, Thymine Containing	Nucleotide	HMDB00079	1.04	0.6586
2'-Deoxyuridine	Pyrimidine Metabolism, Uracil Containing	Nucleotide	HMDB00012	0.68	0.0023
2'-O-Methyluridine	Pyrimidine Metabolism, Uracil Containing	Nucleotide		1.06	0.7953
3-Ureidopropionate	Pyrimidine Metabolism, Uracil Containing	Nucleotide	HMDB00026	1.09	0.5887
5-Methyluridine (Ribothymidine)	Pyrimidine Metabolism, Uracil Containing	Nucleotide	HMDB00884	0.99	0.8589
Beta-Alanine	Pyrimidine Metabolism, Uracil Containing	Nucleotide	HMDB00056	1.16	0.3441
N-Acetyl-Beta-Alanine	Pyrimidine Metabolism, Uracil Containing	Nucleotide		0.97	0.8090
Pseudouridine	Pyrimidine Metabolism, Uracil Containing	Nucleotide	HMDB00767	1.19	0.0629
Uracil	Pyrimidine Metabolism, Uracil Containing	Nucleotide	HMDB00300	1.17	0.6905

Uridine	Pyrimidine Metabolism, Uracil Containing	Nucleotide	HMDB00296	0.93	0.3329
4-Hydroxyphenylacetylglutamine	Acetylated Peptides	Peptide		1.62	0.2601
Phenylacetylcarnitine	Acetylated Peptides	Peptide		1.45	0.1842
Phenylacetylglutamate	Acetylated Peptides	Peptide	HMDB59772	1.80	0.1868
Phenylacetylglutamine	Acetylated Peptides	Peptide	HMDB06344	1.52	0.0325
Phenylacetylglycine	Acetylated Peptides	Peptide	HMDB00821	1.64	0.0950
Isoleucylglycine	Dipeptide	Peptide	HMDB28907	0.98	0.9564
Prolylglycine	Dipeptide	Peptide	HMDB11178	0.86	0.6875
Adpsgegdfxaegggvr*	Fibrinogen Cleavage Peptide	Peptide		Excluded	Excluded
Dsgegdfxaegggvr*	Fibrinogen Cleavage Peptide	Peptide		Excluded	Excluded
Gamma-Glutamyl-2-Aminobutyrate	Gamma-Glutamyl Amino Acid	Peptide		0.71	0.0256
Gamma-Glutamyl-Alpha-Lysine	Gamma-Glutamyl Amino Acid	Peptide		1.02	0.8090
Gamma-Glutamyl-Epsilon-Lysine	Gamma-Glutamyl Amino Acid	Peptide	HMDB03869	0.98	0.8731
Gamma-Glutamylalanine	Gamma-Glutamyl Amino Acid	Peptide	HMDB29142	0.87	0.5899
Gamma-Glutamylglutamate	Gamma-Glutamyl Amino Acid	Peptide	HMDB11737	1.12	0.4317
Gamma-Glutamylglutamine	Gamma-Glutamyl Amino Acid	Peptide	HMDB11738	1.08	0.3929
Gamma-Glutamylglycine	Gamma-Glutamyl Amino Acid	Peptide	HMDB11667	1.08	0.4686
Gamma-Glutamylhistidine	Gamma-Glutamyl Amino Acid	Peptide		1.02	0.8353
Gamma-Glutamylisoleucine*	Gamma-Glutamyl Amino Acid	Peptide	HMDB11170	1.13	0.3332
Gamma-Glutamylleucine	Gamma-Glutamyl Amino Acid	Peptide	HMDB11171	1.01	0.9173
Gamma-Glutamylmethionine	Gamma-Glutamyl Amino Acid	Peptide	HMDB29155	1.03	0.8110
Gamma-Glutamylphenylalanine	Gamma-Glutamyl Amino Acid	Peptide	HMDB00594	1.07	0.3441
Gamma-Glutamylthreonine	Gamma-Glutamyl Amino Acid	Peptide	HMDB29159	1.01	0.9248
Gamma-Glutamyltryptophan	Gamma-Glutamyl Amino Acid	Peptide	HMDB29160	0.86	0.0430
Gamma-Glutamyltyrosine	Gamma-Glutamyl Amino Acid	Peptide	HMDB11741	0.98	0.8260
Gamma-Glutamylvaline	Gamma-Glutamyl Amino Acid	Peptide	HMDB11172	1.12	0.2577
N-Methylpipecolate	Bacterial/Fungal	Xenobiotics		1.44	0.3009
Tartronate (Hydroxymalonate)	Bacterial/Fungal	Xenobiotics	HMDB35227	1.04	0.8961
2-Ethylphenylsulfate	Benzoate Metabolism	Xenobiotics		Excluded	Excluded
2-Hydroxyhippurate (Salicylurate)	Benzoate Metabolism	Xenobiotics	HMDB00840	1.98	0.5802
3-(3-Hydroxyphenyl)Propionate	Benzoate Metabolism	Xenobiotics	HMDB00375	0.76	0.5779
Sulfate	Benzoate Metabolism	Xenobiotics		0.61	0.2779

3-Hydroxyhippurate	Benzoate Metabolism	Xenobiotics	HMDB06116		0.78	0.6681
3-Methoxycatechol Sulfate (1)	Benzoate Metabolism	Xenobiotics			0.71	0.3554
3-Methyl Catechol Sulfate (1)	Benzoate Metabolism	Xenobiotics			0.66	0.2256
3-Methyl Catechol Sulfate (2)	Benzoate Metabolism	Xenobiotics		Excluded	Excluded	
3-Phenylpropionate (Hydrocinnamate)	Benzoate Metabolism	Xenobiotics	HMDB00764		0.82	0.4175
4-Ethylphenylsulfate	Benzoate Metabolism	Xenobiotics			1.59	0.5959
4-Hydroxyhippurate	Benzoate Metabolism	Xenobiotics	HMDB13678		1.36	0.3109
4-Methylcatechol Sulfate	Benzoate Metabolism	Xenobiotics			1.10	0.7718
4-Vinylphenol Sulfate	Benzoate Metabolism	Xenobiotics	HMDB04072		0.90	0.7839
Benzoate	Benzoate Metabolism	Xenobiotics	HMDB01870		0.74	0.0001
Catechol Sulfate	Benzoate Metabolism	Xenobiotics	HMDB59724		0.86	0.6001
Hippurate	Benzoate Metabolism	Xenobiotics	HMDB00714		1.06	0.8854
Methyl-4-Hydroxybenzoate Sulfate	Benzoate Metabolism	Xenobiotics			2.05	0.6031
O-Cresol Sulfate	Benzoate Metabolism	Xenobiotics			0.53	0.0657
O-Methylcatechol Sulfate	Benzoate Metabolism	Xenobiotics	HMDB60013		0.96	0.8731
P-Cresol Sulfate	Benzoate Metabolism	Xenobiotics	HMDB11635		1.39	0.0657
Propyl 4-Hydroxybenzoate Sulfate	Benzoate Metabolism	Xenobiotics		Excluded	Excluded	
1,2,3-Benzenetriol Sulfate (1)	Chemical	Xenobiotics		Excluded	Excluded	
1,2,3-Benzenetriol Sulfate (2)	Chemical	Xenobiotics			0.83	0.7135
2-Aminophenol Sulfate	Chemical	Xenobiotics	HMDB61116		1.42	0.2624
2-Methoxyresorcinol Sulfate	Chemical	Xenobiotics			1.49	0.7463
3-Acetylphenol Sulfate	Chemical	Xenobiotics			0.73	0.5193
3-Hydroxyindolin-2-One Sulfate	Chemical	Xenobiotics			1.64	0.1081
3-Hydroxypyridine Sulfate	Chemical	Xenobiotics			0.71	0.4156
4-Hydroxychlorothalonil	Chemical	Xenobiotics			0.75	0.0162
4-Methylbenzenesulfonate	Chemical	Xenobiotics			0.71	0.0382
6-Hydroxyindole Sulfate	Chemical	Xenobiotics			1.28	0.2334
Dimethyl Sulfone	Chemical	Xenobiotics	HMDB04983		1.62	0.4968
Dimethyl Sulfoxide (Dmso)	Chemical	Xenobiotics	HMDB02151		0.94	0.8338
Ectoine	Chemical	Xenobiotics			1.57	0.3009
Edta	Chemical	Xenobiotics	HMDB15109		1.07	0.1324
Ethyl Glucuronide	Chemical	Xenobiotics	HMDB10325	Excluded	Excluded	
Iminodiacetate (Ida)	Chemical	Xenobiotics	HMDB11753		1.22	0.0000

Lanthionine	Chemical	Xenobiotics		1.37	0.5490
O-Sulfo-L-Tyrosine	Chemical	Xenobiotics		1.18	0.1859
Perfluorooctanesulfonic Acid (Pfos)	Chemical	Xenobiotics	HMDB59586	0.95	0.8540
Succinimide	Chemical	Xenobiotics		0.97	0.8353
Sulfate*	Chemical	Xenobiotics	HMDB01448	1.02	0.7475
Thioproline	Chemical	Xenobiotics		1.19	0.0053
1-Hydroxy-2-Naphthalenecarboxylate	Drug	Xenobiotics		Excluded	Excluded
2-Acetamidophenol Sulfate	Drug	Xenobiotics		2.06	0.1545
2-Hydroxyacetaminophen Sulfate*	Drug	Xenobiotics		Excluded	Excluded
2-Hydroxyibuprofen	Drug	Xenobiotics	HMDB60920	Excluded	Excluded
Glucuronide*	Drug	Xenobiotics		Excluded	Excluded
2-Methoxyacetaminophen Sulfate*	Drug	Xenobiotics		Excluded	Excluded
3-(Cystein-S-Yl)Acetaminophen*	Drug	Xenobiotics		Excluded	Excluded
3-(N-Acetyl-L-Cystein-S-Yl) Acetaminophen	Drug	Xenobiotics		Excluded	Excluded
3-Hydroxyquinine	Drug	Xenobiotics	HMDB01091	Excluded	Excluded
4-Acetamidophenol	Drug	Xenobiotics	HMDB01859	Excluded	Excluded
4-Acetamidophenylglucuronide	Drug	Xenobiotics	HMDB10316	Excluded	Excluded
4-Acetaminophen Sulfate	Drug	Xenobiotics	HMDB59911	9.50	0.2334
4-Acetylphenol Sulfate	Drug	Xenobiotics		1.00	0.9921
4-Aminophenol Sulfate (2)	Drug	Xenobiotics		Excluded	Excluded
4-Hydroxycoumarin	Drug	Xenobiotics		1.18	0.6875
Allopurinol	Drug	Xenobiotics		Excluded	Excluded
Amoxicillin	Drug	Xenobiotics	HMDB15193	Excluded	Excluded
Atenolol	Drug	Xenobiotics	HMDB01924	Excluded	Excluded
Baclofen	Drug	Xenobiotics	HMDB14327	Excluded	Excluded
Candesartan	Drug	Xenobiotics	HMDB14934	Excluded	Excluded
Carboxyibuprofen	Drug	Xenobiotics	HMDB60564	Excluded	Excluded
Cetirizine	Drug	Xenobiotics	HMDB05032	Excluded	Excluded
Desmethylnaproxen	Drug	Xenobiotics	HMDB13989	Excluded	Excluded
Desmethylnaproxen Sulfate	Drug	Xenobiotics		Excluded	Excluded
Dexlansoprazole	Drug	Xenobiotics		Excluded	Excluded
Diclofenac	Drug	Xenobiotics	HMDB14724	Excluded	Excluded

Diltiazem	Drug	Xenobiotics	HMDB14487	Excluded	Excluded	
Escitalopram	Drug	Xenobiotics	HMDB05028	Excluded	Excluded	
Fluoxetine	Drug	Xenobiotics		Excluded	Excluded	
Furosemide	Drug	Xenobiotics	HMDB01933	Excluded	Excluded	
Gabapentin	Drug	Xenobiotics	HMDB05015	Excluded	Excluded	
Hydroquinone Sulfate	Drug	Xenobiotics	HMDB02434		1.20	0.6001
Hydroxypioglitazone (M-Iv)	Drug	Xenobiotics		Excluded	Excluded	
Ibuprofen	Drug	Xenobiotics	HMDB01925	Excluded	Excluded	
Ibuprofen Acyl Glucuronide	Drug	Xenobiotics		Excluded	Excluded	
Ketopioglitazone	Drug	Xenobiotics		Excluded	Excluded	
Lamotrigine	Drug	Xenobiotics	1DB14695,HMDB05(	Excluded	Excluded	
Levetiracetam	Drug	Xenobiotics		Excluded	Excluded	
Lidocaine	Drug	Xenobiotics	HMDB14426	Excluded	Excluded	
Lisinopril	Drug	Xenobiotics		Excluded	Excluded	
Metformin	Drug	Xenobiotics	HMDB01921	Excluded	Excluded	
N-Ethylglycinexylidide	Drug	Xenobiotics	HMDB60656	Excluded	Excluded	
Naproxen	Drug	Xenobiotics	HMDB01923	Excluded	Excluded	
Norfluoxetine	Drug	Xenobiotics	HMDB60551	Excluded	Excluded	
O-Desmethyltramadol	Drug	Xenobiotics	HMDB60997	Excluded	Excluded	
O-Desmethyltramadol Glucuronide	Drug	Xenobiotics		Excluded	Excluded	
O-Desmethylvenlafaxine	Drug	Xenobiotics	HMDB60532	Excluded	Excluded	
Olmesartan	Drug	Xenobiotics	HMDB14420	Excluded	Excluded	
Omeprazole	Drug	Xenobiotics	HMDB01913	Excluded	Excluded	
Oxypurinol	Drug	Xenobiotics	HMDB00786	Excluded	Excluded	
Pantoprazole	Drug	Xenobiotics	HMDB05017	Excluded	Excluded	
Pioglitazone	Drug	Xenobiotics	HMDB15264	Excluded	Excluded	
Pivaloylcarnitine (C5)	Drug	Xenobiotics	HMDB41993	Excluded	Excluded	
Quinine	Drug	Xenobiotics		Excluded	Excluded	
Ranitidine	Drug	Xenobiotics	HMDB01930	Excluded	Excluded	
Rivaroxaban	Drug	Xenobiotics		Excluded	Excluded	
Rosuvastatin	Drug	Xenobiotics	HMDB15230	Excluded	Excluded	
Salicylate	Drug	Xenobiotics	HMDB01895		1.31	0.8353
Salicyluric Glucuronide*	Drug	Xenobiotics			1.44	0.6595

Sertraline	Drug	Xenobiotics	HMDB05010	Excluded	Excluded
Sitagliptin	Drug	Xenobiotics		Excluded	Excluded
Tramadol	Drug	Xenobiotics	HMDB14339	Excluded	Excluded
Valproate	Drug	Xenobiotics	HMDB01877	Excluded	Excluded
Venlafaxine	Drug	Xenobiotics	HMDB05016	Excluded	Excluded
Warfarin	Drug	Xenobiotics	HMDB01935	Excluded	Excluded
2,3-Dihydroxyisovalerate	Food Component/Plant	Xenobiotics	HMDB12141	0.60	0.4377
2-Keto-3-Deoxy-Gluconate	Food Component/Plant	Xenobiotics	HMDB01353	0.81	0.3297
2-Piperidinone	Food Component/Plant	Xenobiotics	HMDB11749	2.15	0.2741
3,4-Methyleneheptanoate	Food Component/Plant	Xenobiotics		0.96	0.9069
4-Allylphenol Sulfate	Food Component/Plant	Xenobiotics		0.80	0.6465
4-Vinylguaiacol Sulfate	Food Component/Plant	Xenobiotics		1.06	0.8978
Acesulfame	Food Component/Plant	Xenobiotics	HMDB33585	Excluded	Excluded
Beta-Guanidinopropanoate	Food Component/Plant	Xenobiotics	HMDB13222	1.04	0.9163
Cinnamoylglycine	Food Component/Plant	Xenobiotics	HMDB11621	0.87	0.6905
Daidzein Sulfate (2)	Food Component/Plant	Xenobiotics		Excluded	Excluded
Dihydroferulic Acid	Food Component/Plant	Xenobiotics		0.81	0.6372
Ergothioneine	Food Component/Plant	Xenobiotics	HMDB03045	0.61	0.0178
Erythritol	Food Component/Plant	Xenobiotics	HMDB02994	1.08	0.6465
Eugenol Sulfate	Food Component/Plant	Xenobiotics		2.25	0.4968
Ferulic Acid 4-Sulfate	Food Component/Plant	Xenobiotics	HMDB29200	0.98	0.9592
Furaneol Sulfate	Food Component/Plant	Xenobiotics		1.31	0.6601
Gluconate	Food Component/Plant	Xenobiotics	HMDB00625	1.23	0.2826
Homostachydrine*	Food Component/Plant	Xenobiotics	HMDB33433	0.98	0.9578
Indolin-2-One	Food Component/Plant	Xenobiotics		1.54	0.0937
Isoeugenol Sulfate	Food Component/Plant	Xenobiotics		Excluded	Excluded
Beta)	Food Component/Plant	Xenobiotics		0.59	0.3193
N-(2-Furoyl)Glycine	Food Component/Plant	Xenobiotics	HMDB00439	0.77	0.6465
N-Acetylalliin	Food Component/Plant	Xenobiotics		Excluded	Excluded
Naringenin 7-Glucuronide	Food Component/Plant	Xenobiotics		Excluded	Excluded
Piperine	Food Component/Plant	Xenobiotics	HMDB29377	1.10	0.8761
Pyrraline	Food Component/Plant	Xenobiotics	HMDB33143	1.01	0.9670
Quinate	Food Component/Plant	Xenobiotics	HMDB03072	0.69	0.2009

S-Allylcysteine	Food Component/Plant	Xenobiotics	HMDB34323	Excluded	Excluded
Saccharin	Food Component/Plant	Xenobiotics	HMDB29723	4.57	0.2256
Stachydrine	Food Component/Plant	Xenobiotics	HMDB04827	0.96	0.9420
Tartarate	Food Component/Plant	Xenobiotics	HMDB00956	0.58	0.3009
Theanine	Food Component/Plant	Xenobiotics	HMDB34365	0.70	0.4425
Thymol Sulfate	Food Component/Plant	Xenobiotics	HMDB01878	0.79	0.7876
Umbelliferone Sulfate	Food Component/Plant	Xenobiotics		2.36	0.5066
3-Hydroxycotinine Glucuronide	Tobacco Metabolite	Xenobiotics	HMDB01204	Excluded	Excluded
Cotinine	Tobacco Metabolite	Xenobiotics	HMDB01046	Excluded	Excluded
Cotinine N-Oxide	Tobacco Metabolite	Xenobiotics	HMDB01411	Excluded	Excluded
Hydroxycotinine	Tobacco Metabolite	Xenobiotics	HMDB01390	Excluded	Excluded
Norcotinine	Tobacco Metabolite	Xenobiotics	HMDB01297	Excluded	Excluded
1,3,7-Trimethylurate	Xanthine Metabolism	Xenobiotics	HMDB02123	0.73	0.3361
1,3-Dimethylurate	Xanthine Metabolism	Xenobiotics	HMDB01857	0.74	0.1509
1,7-Dimethylurate	Xanthine Metabolism	Xenobiotics	HMDB11103	0.79	0.2334
1-Methylurate	Xanthine Metabolism	Xenobiotics	HMDB03099	0.83	0.5236
1-Methylxanthine	Xanthine Metabolism	Xenobiotics	HMDB10738	0.59	0.0061
3,7-Dimethylurate	Xanthine Metabolism	Xenobiotics	HMDB01982	0.96	0.9069
3-Methylxanthine	Xanthine Metabolism	Xenobiotics	HMDB01886	1.03	0.9420
Methyluracil	Xanthine Metabolism	Xenobiotics	HMDB04400	0.90	0.7207
5-Acetylamino-6-Formylamino-3-Methyluracil	Xanthine Metabolism	Xenobiotics	HMDB11105	0.91	0.8457
7-Methylurate	Xanthine Metabolism	Xenobiotics	HMDB11107	1.09	0.7617
7-Methylxanthine	Xanthine Metabolism	Xenobiotics	HMDB01991	0.96	0.8978
Caffeic Acid Sulfate	Xanthine Metabolism	Xenobiotics	HMDB41708	0.52	0.0301
Caffeine	Xanthine Metabolism	Xenobiotics	HMDB01847	0.57	0.0096
Paraxanthine	Xanthine Metabolism	Xenobiotics	HMDB01860	0.69	0.0439
Theobromine	Xanthine Metabolism	Xenobiotics	HMDB02825	0.87	0.6565
Theophylline	Xanthine Metabolism	Xenobiotics	HMDB01889	0.68	0.0255
