# Supplementary material for: Metabolomic changes associated with frontotemporal lobar degeneration syndromes
Source: J Neurol. 2020 Apr 10;267(8):2228–38. doi: 10.1007/s00415-020-09824-1 (PMC7359154; doi:10.1007/s00415-020-09824-1)
Supplement: Supplementary file 2 — Supplementary file2 (PDF 114 kb) [file 415_2020_9824_MOESM2_ESM.pdf]

Subpathway (from local PCA components)	Component 3 loading
Alanine and Aspartate Metabolism Component 1	-0.22
Alanine and Aspartate Metabolism Component 2	0.00
Alanine and Aspartate Metabolism Component 3	0.33
Creatine Metabolism Component 1	-0.09
Creatine Metabolism Component 2	0.00
Glutamate Metabolism Component 1	-0.37
Glutamate Metabolism Component 2	-0.16
Glutamate Metabolism Component 3	-0.06
Glutamate Metabolism Component 4	0.11
Glutathione Metabolism Component 1	-0.36
Glutathione Metabolism Component 2	-0.12
Glycine, Serine and Threonine Metabolism Component 1	**-0.49**
Glycine, Serine and Threonine Metabolism Component 2	-0.32
Glycine, Serine and Threonine Metabolism Component 3	0.17
Glycine, Serine and Threonine Metabolism Component 4	0.38
Guanidino and Acetamido Metabolism Component 1	-0.10
Histidine Metabolism Component 1	-0.34
Histidine Metabolism Component 2	-0.26
Histidine Metabolism Component 3	-0.06
Histidine Metabolism Component 4	0.01
Histidine Metabolism Component 5	0.20
Leucine, Isoleucine and Valine Metabolism Component 1	**-0.48**
Leucine, Isoleucine and Valine Metabolism Component 2	-0.02
Leucine, Isoleucine and Valine Metabolism Component 3	0.00
Leucine, Isoleucine and Valine Metabolism Component 4	0.02
Leucine, Isoleucine and Valine Metabolism Component 5	0.02
Leucine, Isoleucine and Valine Metabolism Component 6	0.09
Leucine, Isoleucine and Valine Metabolism Component 7	0.30
Lysine Metabolism Component 1	**-0.52**
Lysine Metabolism Component 2	-0.19
Lysine Metabolism Component 3	-0.13
Lysine Metabolism Component 4	0.12
Methionine, Cysteine, Sam and Taurine Metabolism Component 1	**-0.48**
Methionine, Cysteine, Sam and Taurine Metabolism Component 2	-0.22
Methionine, Cysteine, Sam and Taurine Metabolism Component 3	-0.06
Methionine, Cysteine, Sam and Taurine Metabolism Component 4	-0.04
Methionine, Cysteine, Sam and Taurine Metabolism Component 5	0.25
Methionine, Cysteine, Sam and Taurine Metabolism Component 6	0.31
Methionine, Cysteine, Sam and Taurine Metabolism Component 7	**0.40**
Phenylalanine Metabolism Component 1	-0.24
Phenylalanine Metabolism Component 2	0.21
Polyamine Metabolism Component 1	0.17
Polyamine Metabolism Component 2	0.21
Tryptophan Metabolism Component 1	**-0.50**

Tryptophan Metabolism Component 2	-0.36
Tryptophan Metabolism Component 3	-0.12
Tryptophan Metabolism Component 4	-0.04
Tryptophan Metabolism Component 5	0.08
Tryptophan Metabolism Component 6	0.30
Tyrosine Metabolism Component 1	-0.30
Tyrosine Metabolism Component 2	-0.02
Tyrosine Metabolism Component 3	0.06
Tyrosine Metabolism Component 4	0.06
Tyrosine Metabolism Component 5	0.06
Tyrosine Metabolism Component 6	0.30
Urea Cycle; Arginine and Proline Metabolism Component 1	**-0.42**
Urea Cycle; Arginine and Proline Metabolism Component 2	-0.32
Urea Cycle; Arginine and Proline Metabolism Component 3	-0.18
Urea Cycle; Arginine and Proline Metabolism Component 4	-0.15
Urea Cycle; Arginine and Proline Metabolism Component 5	-0.08
Urea Cycle; Arginine and Proline Metabolism Component 6	-0.03
Advanced Glycation End-Product Component 1	0.04
Aminosugar Metabolism Component 1	0.24
Disaccharides and Oligosaccharides Component 1	0.22
Fructose, Mannose and Galactose Metabolism Component 1	-0.13
Glycogen Metabolism Component 1	**0.47**
Glycolysis, Gluconeogenesis, and Pyruvate Metabolism Component 1	-0.24
Glycolysis, Gluconeogenesis, and Pyruvate Metabolism Component 2	-0.19
Glycolysis, Gluconeogenesis, and Pyruvate Metabolism Component 3	0.17
Pentose Metabolism Component 1	-0.05
Pentose Metabolism Component 2	0.02
Ascorbate and Aldarate Metabolism Component 1	-0.18
Folate Metabolism Component 1	0.07
Hemoglobin and Porphyrin Metabolism Component 1	-0.14
Hemoglobin and Porphyrin Metabolism Component 2	-0.12
Hemoglobin and Porphyrin Metabolism Component 3	**0.57**
Riboflavin Metabolism Component 1	-0.24
Riboflavin Metabolism Component 2	0.02
Riboflavin Metabolism Component 3	0.05
Tocopherol Metabolism Component 1	-0.03
Vitamin A Metabolism Component 1	-0.05
Oxidative Phosphorylation Component 1	-0.16
Oxidative Phosphorylation Component 2	0.14
Oxidative Phosphorylation Component 3	0.18
Tca Cycle Component 1	-0.19
Tca Cycle Component 2	0.03
Tca Cycle Component 3	0.06
Tca Cycle Component 4	0.11
androgenic Steroids Component 1	-0.25
Carnitine Metabolism Component 1	-0.12

Carnitine Metabolism Component 2	-0.08
Carnitine Metabolism Component 3	0.32
Carnitine Metabolism Component 4	0.37
Ceramides Component 1	0.03
Corticosteroids Component 1	-0.27
Diacylglycerol Component 1	-0.15
Diacylglycerol Component 2	0.10
Diacylglycerol Component 3	0.20
Endocannabinoid Component 1	0.34
Fatty Acid Metabolism (Acyl Glutamine) Component 1	-0.24
Fatty Acid Metabolism (Acyl Glutamine) Component 2	-0.17
Fatty Acid Metabolism (Also Bcaa Metabolism) Component 1	0.12
Fatty Acid Metabolism (Also Bcaa Metabolism) Component 2	0.14
Fatty Acid Metabolism (Also Bcaa Metabolism) Component 3	0.19
Fatty Acid Metabolism (Also Bcaa Metabolism) Component 4	0.27
Fatty Acid Metabolism (Also Bcaa Metabolism) Component 5	0.33
Fatty Acid Metabolism(Acyl Carnitine) Component 1	0.38
Fatty Acid Synthesis Component 1	-0.33
Fatty Acid, Amide Component 1	-0.11
Fatty Acid, Amino Component 1	0.10
Fatty Acid, Branched Component 1	-0.34
Fatty Acid, Branched Component 2	-0.34
Fatty Acid, Branched Component 3	-0.16
Fatty Acid, Branched Component 4	-0.03
Fatty Acid, Branched Component 5	-0.03
Fatty Acid, Branched Component 6	0.36
Fatty Acid, Dicarboxylate Component 1	-0.28
Fatty Acid, Dihydroxy Component 1	-0.37
Fatty Acid, Dihydroxy Component 2	-0.25
Fatty Acid, Dihydroxy Component 3	-0.18
Fatty Acid, Dihydroxy Component 4	0.05
Fatty Acid, Dihydroxy Component 5	0.16
Fatty Acid, Monohydroxy Component 1	**-0.54**
Fatty Acid, Monohydroxy Component 2	0.24
Glycerolipid Metabolism Component 1	0.04
Inositol Metabolism Component 1	0.29
Ketone Bodies Component 1	-0.03
Ketone Bodies Component 2	0.15
Long Chain Fatty Acid Component 1	-0.39
Long Chain Fatty Acid Component 2	-0.02
Long Chain Fatty Acid Component 3	0.03
Long Chain Fatty Acid Component 4	0.09
Long Chain Fatty Acid Component 5	0.14
Lysophospholipid Component 1	**0.40**
Lysoplasmalogen Component 1	0.02
Lysoplasmalogen Component 2	0.12

Medium Chain Fatty Acid Component 1	-0.05
Mevalonate Metabolism Component 1	-0.17
Mevalonate Metabolism Component 2	-0.06
Mevalonate Metabolism Component 3	**0.52**
Monoacylglycerol Component 1	-0.05
Monoacylglycerol Component 2	-0.01
Monoacylglycerol Component 3	0.00
Monoacylglycerol Component 4	0.13
Monoacylglycerol Component 5	**0.41**
Phosphatidylcholine (Pc) Component 1	-0.05
Phosphatidylcholine (Pc) Component 2	-0.03
Phosphatidylcholine (Pc) Component 3	**0.43**
Phosphatidylethanolamine (Pe) Component 1	0.03
Phosphatidylinositol (Pi) Component 1	**0.58**
Phosphatidylserine (Ps) Component 1	-0.19
Phosphatidylserine (Ps) Component 2	-0.05
Phosphatidylserine (Ps) Component 3	**0.52**
Phospholipid Metabolism Component 1	0.17
Phospholipid Metabolism Component 2	0.19
Phospholipid Metabolism Component 3	0.31
Plasmalogen Component 1	-0.31
Plasmalogen Component 2	-0.06
Plasmalogen Component 3	0.13
Polyunsaturated Fatty Acid (N3 and N6) Component 1	-0.10
Pregnenolone Steroids Component 1	-0.04
Pregnenolone Steroids Component 2	0.04
Primary Bile Acid Metabolism Component 1	-0.07
Progestin Steroids Component 1	-0.11
Progestin Steroids Component 2	0.01
Progestin Steroids Component 3	0.02
Progestin Steroids Component 4	0.03
Progestin Steroids Component 5	0.09
Progestin Steroids Component 6	0.12
Secondary Bile Acid Metabolism Component 1	**-0.40**
Secondary Bile Acid Metabolism Component 2	-0.07
Secondary Bile Acid Metabolism Component 3	-0.04
Secondary Bile Acid Metabolism Component 4	-0.03
Secondary Bile Acid Metabolism Component 5	-0.03
Secondary Bile Acid Metabolism Component 6	0.15
Secondary Bile Acid Metabolism Component 7	0.20
Secondary Bile Acid Metabolism Component 8	**0.40**
Sphingolipid Metabolism Component 1	-0.05
Sphingolipid Metabolism Component 2	0.20
Sterol Component 1	-0.27
Sterol Component 2	0.15
Purine Metabolism, (Hypo)Xanthine/Inosine Containing Component 1	0.21

Purine Metabolism, (Hypo)Xanthine/Inosine Containing Component 2	0.29
Purine Metabolism, Adenine Containing Component 1	0.13
Purine Metabolism, Guanine Containing Component 1	0.18
Purine Metabolism, Guanine Containing Component 2	0.23
Pyrimidine Metabolism, Cytidine Containing Component 1	-0.08
Pyrimidine Metabolism, Orotate Containing Component 1	0.16
Pyrimidine Metabolism, Thymine Containing Component 1	-0.27
Pyrimidine Metabolism, Thymine Containing Component 2	-0.01
Pyrimidine Metabolism, Thymine Containing Component 3	-0.01
Pyrimidine Metabolism, Thymine Containing Component 4	0.07
Pyrimidine Metabolism, Uracil Containing Component 1	0.08
Acetylated Peptides Component 1	-0.19
Dipeptide Component 1	-0.38
Dipeptide Component 2	-0.27
Dipeptide Component 3	0.12
Dipeptide Component 4	0.20
Fibrinogen Cleavage Peptide Component 1	-0.05
Gamma-Glutamyl Amino Acid Component 1	-0.22
Gamma-Glutamyl Amino Acid Component 2	-0.10
Gamma-Glutamyl Amino Acid Component 3	-0.04
Gamma-Glutamyl Amino Acid Component 4	0.13
Gamma-Glutamyl Amino Acid Component 5	0.15
Gamma-Glutamyl Amino Acid Component 6	0.18
Gamma-Glutamyl Amino Acid Component 7	0.28
